# The Bourque distances for mutation trees of cancers

**DOI:** 10.1186/s13015-021-00188-3

**Published:** 2021-06-10

**Authors:** Katharina Jahn, Niko Beerenwinkel, Louxin Zhang

**Affiliations:** 1 Department of Biosystems Science and Engineering, ETH Zurich, Basel, Switzerland; 2 SIB Swiss Institute of Bioinformatics, Basel, Switzerland; 3 Department of Mathematics and Computational Biology Programme, National University of Singapore, 119076 Singapore, Singapore

**Keywords:** Labeled phylogenetic trees, Mutation trees, The nearest neighbor interchange distance, Robinson−Foulds distance, Bourque distances

## Abstract

****Background**:**

Mutation trees are rooted trees in which nodes are of arbitrary degree and labeled with a mutation set. These trees, also referred to as clonal trees, are used in computational oncology to represent the mutational history of tumours. Classical tree metrics such as the popular Robinson–Foulds distance are of limited use for the comparison of mutation trees. One reason is that mutation trees inferred with different methods or for different patients often contain different sets of mutation labels.

****Results**:**

We generalize the Robinson–Foulds distance into a set of distance metrics called Bourque distances for comparing mutation trees. We show the basic version of the Bourque distance for mutation trees can be computed in linear time. We also make a connection between the Robinson–Foulds distance and the nearest neighbor interchange distance.

**Supplementary Information:**

The online version contains supplementary material available at 10.1186/s13015-021-00188-3.

## Background

Trees have been used in biology to model the evolution of species, genes and cancer cells [1–3]; to represent the secondary structures of RNA molecules and to classify cell types, to name just a few uses [4, 5]. A fundamental issue arising from these applications of trees is how to quantitatively compare tree models that are inferred by different methods or from different data. A number of tree metrics have been proposed for comparisons, including the Robinson–Foulds (RF) [6–8], nearest-neighbor interchange (NNI) [7, 9] and triple(t) distances [10] for phylogenetic trees; gene duplication, gene loss and reconciliation costs [11, 12] for gene and species trees; and the tree-edit distances [5, 13, 14] for tree models of secondary RNA structures, etc. [15–19].

With advances in next-generation sequencing and single-cell sequencing technologies, a large amount of genomic data is now available for identifying tumour subclones and inferring their evolutionary relationships. The most common representation of these relationships are mutation trees, also known as clonal trees, which encode the (partial) temporal order in which mutations were acquired. Formally, a mutation tree on a finite set of mutations $\Gamma$ is a rooted tree *T* with *k* nodes and a partition of $\Gamma$ into *k* disjoint non-empty parts $P_i$ so that each $P_i$ is assigned as the label of a node of *T* [2, 20]. A large number of computational approaches for reconstructing mutation trees from bulk sequencing data [21–25], single-cell sequencing data [26–29], or a combination of both [30, 31] have been developed over the last years. Unlike phylogenetic trees, mutation trees inferred with these methods will not only differ in their topology but may also be defined on different sets of mutations. The latter happens in the comparison of methods using different data (e. g. single-cell vs. bulk) or divergent criteria for mutation calling. For that reason, classical tree distance measures are not immediately applicable to mutation trees. Instead novel measures have recently been developed [32–37], but no standard approach for mutation tree comparison has yet emerged. Instead, shortcomings of some of these measures such as the inability to resolve major differences between trees have recently been demonstrated [34]. Additionally, computing the distances between two mutation trees takes at least quadratic time for each of these measures.

Here, we generalize the Robinson–Foulds metric, a classic distance measure for unrooted trees, for the comparison of mutation trees. This metric is based on the so-called (edge) contraction and decontraction operations introduced by Bourque for leaf-labeled unrooted trees in a study of Steiner trees [6]. A contraction on an edge (*u*.*v*) of a tree *T* is an operation that transforms *T* into a new tree by shrinking (*u*, *v*) into a single node. The decontraction operation is the reverse of contraction. Robinson and Foulds independently adopted the contraction and decontraction to define a metric of unrooted labeled trees, where there is a finite set *S* and a partition of *S* into disjoint parts (some of which may be empty) so that nodes with a degree of at most 2 are each labeled with a unique non-empty part, and nodes with a degree of at least 3 are labeled with either a unique non-empty part or an empty part. They defined a metric, now called the Robinson–Foulds (RF) distance, by which the distance between two unrooted labeled trees is the minimum number of contraction or decontraction operations that are necessary to transform one into another [8]. The RF distance is equal to the number of edge-induced partitions that are not shared between the two trees and thus is computed in linear time [38].

Although the RF distance is popular in phylogenetic analysis, it is not robust when applied to the comparison of mutation trees with different sets of mutations, as it is simply equal to the total number of edges in the trees and thus fails to capture any topological similarity between the trees.

In this paper, by generalizing the RF distance, we propose a collection of distance measures to measure the topological dissimilarity between unrooted (resp. rooted) labeled trees with different label sets. We also apply these measures to simulated and real tumour mutation trees. To set our distances apart from another recently introduced generalised RF distance that is based on a node flip operation [33], we refer to our generalisations as Bourque distances, as they are closely related to the edge contraction and decontraction operations introduced by Bourque for leaf-labeled unrooted trees [6]. They are also shown to be related to the NNI distance [7]. Unlike previous measures proposed for the comparison of mutation trees, the Bourque distances are metrics and the basic version can be computed in linear time.

The rest of this paper is divided into seven sections. "Concepts and notation" section introduces basic concepts and the notation that will be used. In "Metrics for labeled trees", we present a connection between the NNI distance and the RF distance for both phylogenetic and arbitrary trees that are unrooted and labeled. In "Generalizations of the RF distance for labeled trees on different label sets", we generalize the RF distance into the Bourque distances for unrooted labeled trees. In "The Bourque distances for mutation trees", we define the Bourque distances for mutation trees. In "Comparison of eight distance measures on rooted labeled trees", we examine the relationships among the distance measures proposed in [34, 35, 37] and the Bourque distances on rooted 7-node trees and on random rooted trees with 30 nodes. In "Applications to mutation trees", we computed the Bourque distances on two sets of mutation trees. "Conclusions" section concludes the study with a few remarks.

## Concepts and notation

A (unrooted) tree is an acyclic graph. A rooted tree is a directed tree with a designated root node $\rho$ in which the edges are oriented away from $\rho$ and there is a unique directed path from $\rho$ to every other node.

For a tree or rooted tree *T*, the nodes, leaves and edges are denoted *V*(*T*), $\mathrm{Leaf}(T)$ and *E*(*T*), respectively. Let $u\in V(T)$. The degree of *u* is the number of edges incident to it, where edge orientation is ignored if *T* is rooted. In a rooted tree, non-root nodes with a degree of one are called the leaves; non-leaf nodes are called internal nodes. One or more edges may leave an internal node, but exactly one edge enters every node that is not the root. An internal edge is an edge between two internal nodes.

Let $u, v\in V(T)$. The node *v* is called a *child* of *u* and *u* is called the parent of *v* if $(u, v)\in E(T)$. In general, *v* is a descendant of *u* and *u* is an ancestor of *v* if the unique path from the tree root to *v* contains *u*. We use $C_T(u)$, $A_T(u)$ and $D_T(u)$ to denote the set of all children, ancestors and descendants of *u* in *T*, respectively. Note that $u\not \in A_T(u)$ and $u\not \in D_T(u)$.

A star tree is a tree that contains only one non-leaf node, which is called the center of the tree. A rooted star tree is a rooted tree in which all except for the root are leaves.

A line tree is a tree in which every internal node is of degree 2. A rooted line tree is the tree obtained by rooting a line tree at a leaf.

A tree is binary if every internal node is of degree 3. A rooted tree is binary if the root is of degree 2 and every other internal node is of degree 3. A (resp. rooted) caterpillar tree is a binary tree in which each internal node is adjacent to one or two leaves.

Let *X* be a finite set. A phylogenetic tree (resp. rooted phylogenetic tree) *T* on *X* is a binary (resp. rooted) tree where the leaves are uniquely labeled with the elements of *X*, the taxon set. It is labeled if there is a set *I* that is disjoint from *X* and a labeling function $\ell : V(T)\setminus \mathrm{Leaf}(T) \rightarrow I$ such that each *u* of $V(T)\setminus \mathrm{Leaf}(T)$ is labeled with $\ell (u)$ and $\ell (u)\cap \ell (v) = \emptyset$ for all $u\ne v \in V(T) \setminus \mathrm{Leaf}(T)$. If $\ell$ is a one-to-one function, *T* is said to be uniquely labeled or 1-labeled. In a labeled phylogenetic tree, the label set for the internal nodes and the taxon set for the leaves are distinct and thus are not interchangeable.

A tree (resp. rooted tree) *T* with *n* nodes is labeled if there is a finite set *M* and a labeling function $\ell : V(T)\rightarrow 2^M$ satisfying $\cup _{v\in V(T)} \ell (v)=M$ and $\ell (v)\ne \emptyset$ for $v\in V(T)$ so that *f*(*v*) is assigned as the label of *v*, where $2^M$ denotes the collection of subsets of *M*. Furthermore, if $\ell (v)$ contains exactly one element for each node *v*, we say *T* is 1-labeled. Here, *M* is called the label set of *T*.

A mutation tree on a set *M* of mutations is a rooted labeled tree that has *M* as the label set.

## Metrics for labeled trees

For convenience, we will introduce new metrics on the space of 1-labeled trees and then generalize them to the space of mutation trees later.

### NNIs on labeled phylogenetic trees

**Fig. 1:**
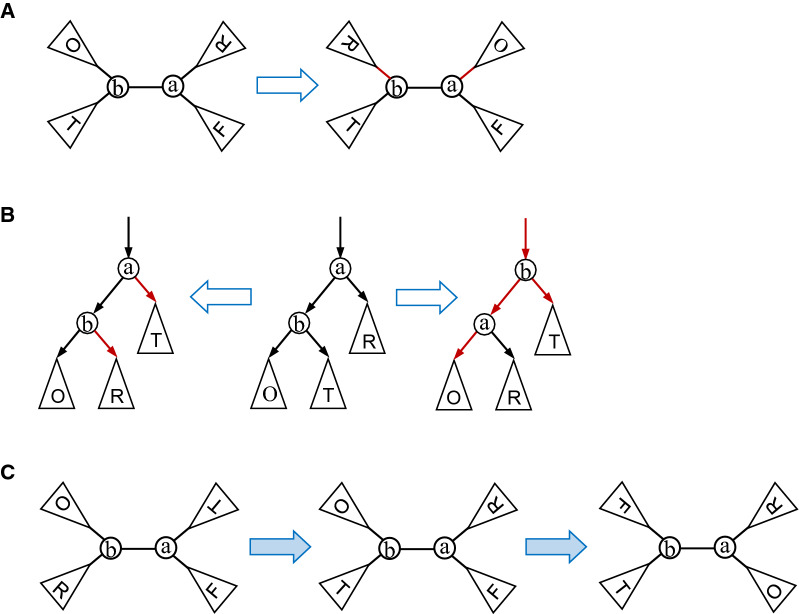
Illustration of the NNI operation on phylogenetic trees. **A** In a phylogenetic tree, an NNI operation on an internal edge (*a*, *b*) first selects two edges (*a*, *x*) and (*b*, *y*) that are, respectively, incident to *a* and *b* such that $(a, x)\ne (a, b)\ne (y, b)$; it then rewires them to the opposite end so that (*a*, *y*) and (*b*, *x*) are the two edges in the resulting tree (red). Since *a* and *b* are labeled differently, a unrooted tree can be transformed into one of four possible trees in one NNI. **B** In a rooted phylogenetic tree *T*, an NNI operation on an internal edge (*a*, *b*) (where *b* is a child of *a*) transforms *T* by either (i) selecting two edges (*a*, *x*) and (*b*, *y*) that leave from *a* and *b*, respectively, and replacing them with (*a*, *y*) and (*b*, *x*) (left), where $x\ne b$, or (ii) selecting an edge (*b*, *y*) leaving from *b* and replacing the unique edge (*z*, *a*) that enters *a*, (*a*, *b*) and (*b*, *y*) with (*z*, *b*), (*b*, *a*) and (*a*, *y*) (right), respectively. A rooted tree can be transformed into four different trees in one NNI. **C** An illustration of the interchange of two labels of the ends of an internal edge in two NNIs in an 1-labeled phylogenetic tree

The NNI operation (Fig. 1A) and NNI distance were originally introduced for unrooted phylogenetic trees [7]. It is known that any binary phylogenetic tree can be transformed into another in $n\log n +2n-4$ NNIs at most [39]. The NNI operation for rooted phylogenetic trees is given in Fig. 1B. Since the NNI operation does never interchange the labels of internal nodes and of leaves, Proposition 1 is simple, but as far as we know, it has never appeared in literature.

#### Proposition 1

In the space of binary (resp. rooted) phylogenetic trees where the internal nodes are 1-labeled, any tree can be transformed into another.

#### Proof

This follows from the fact that two NNIs on an internal edge (*a*, *b*) are enough to exchange the labels of *a* and *b* (Fig. 1C). A similar fact is also true for binary rooted phylogenetic trees.

### Generalized NNI on 1-labeled trees

An arbitrary tree with *n* nodes can have at least 1 and at most $n-2$ internal nodes of degree $\ge 2$. To transform a 1-labeled tree into any other with the same number of nodes on the same label set, we define the generalized NNI (gNNI) operation as follows.

#### Definition 1

Let *T* be a 1-labeled tree and $e=(a, b)\in E(T)$. A gNNI on *e* is an operation that transforms *T* into a new tree *S* by (i) selecting a subset $C_a$ and a subset $C_b$ of the edges that are, respectively, incident to *a* and *b* such that $e \not \in C_a\cup C_b$ and then (ii) replacing each edge (*a*, *x*) of $C_a$ with (*b*, *x*) and each edge (*b*, *y*) of $C_b$ with (*a*, *y*).

**Fig. 2:**
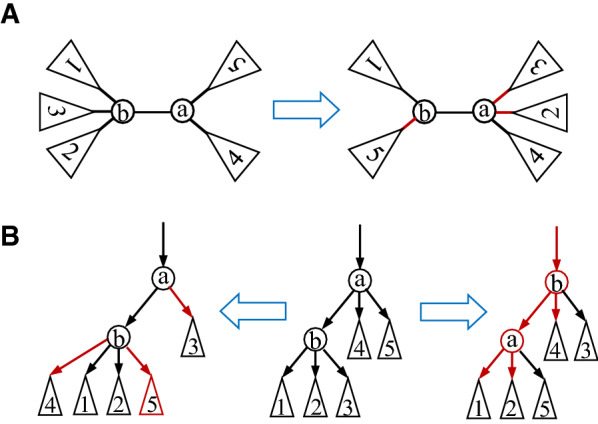
Illustration of the gNNI operation on labeled trees. **A** On a unrooted labeled tree, a gNNI operation on an edge (*a*, *b*) interchanges one or more children of *a* with an arbitrary number of children of *b*. **B** On a rooted labeled tree, a gNNI operation on an edge (*a*, *b*) (where *b* is the child of *a*) not only rewires the selected edges leaving *a* and *b* (left), but also rewires the unique edge entering *a* and *b* simultaneously if necessary (right).

The gNNI operation is illustrated in Fig. 2. Note that if we apply a gNNI operation on an edge $e=(a, b)$ to reconnect all the children of *a* to *b* while keeping the children of *b* unmoved, *a* will become a leaf adjacent to *b* in the resulting tree. An important difference between the gNNI and the NNI is that the gNNI can be applied to any edge, whereas the NNI is defined only on internal edges.

Let *L* be a set of *n* elements. The gNNI graph $G_{\mathrm {gnni}}(L)$ is defined as a graph in which the nodes are all 1-labeled trees with nodes labeled with *L* and two trees are connected by an edge if the two trees are one gNNI apart. The diameter of $G_{\mathrm {gnni}}(L)$ is written as $D(G_{\mathrm {gnni}}(L))$. The distance between two trees $T'$ and $T''$ in the graph is called the *gNNI distance* between them, written as $d_{\mathrm {gnni}}(T', T'')$.

#### Proposition 2

Let *L* be a set of *n* elements. The graph $G_{\mathrm {gnni}}(L)$ has the following properties:

- $$|{V}(G_{\mathrm {gnni}}(L))|=n^{n-2}$$;
- $$G_{\mathrm {gnni}}(L)$$ is connected;
- $$n-2 \le D(G_{\mathrm {gnni}}(L))\le 2n-4$$

#### Proof

The first property is the Cayley formula on the count of 1-labeled *n*-node trees. The second property is a consequence of the third that can be proved as follows.

Let $T_1, T_2 \in { V}(G_{\mathrm {gnni}}(L))$. Let $r_1$ and $r_2$ be the two nodes of $T_1$ and $T_2$, respectively, that have the same label. Each *n*-node tree has at least two leaves and therefore $n-2$ internal nodes at most. By applying a gNNI operation on an edge $(r_1, u)$, we can reconnect all the subtrees that each contain exactly one neighbor of *u* to $r_1$, producing a tree in which *u* becomes a leaf adjacent to $r_1$. By continuing to apply the gNNI operation on the edges between $r_1$ and its non-leaf neighbors, we can transform $T_1$ into the star tree centered at $r_1$ in $n-2$ gNNIs at most. In reverse, we can transform the star tree centered at $r_2$ into $T_2$ in $n-2$ gNNIs at most. By combining these two transformations, we transform $T_1$ into $T_2$ by using $2n-4$ gNNIs at most. This proves the upper bound of the third statement.

Let *S* be a line tree where the leaves are labeled with *a* and *b* and let *T* be a 1-labeled star tree centered at the node of the label *a*. The distances between *a* and *b* are $(n-1)$ and 1 in *S* and *T*, respectively. It takes at least $(n-2)$ gNNIs to transform *S* to *T*, as each gNNI can only decrease the distance between *a* and *b* by 1. This proves the lower bound of the third property.

Let *T* be a tree in $G_{\mathrm {gnni}}(L)$. We use *d*(*u*, *v*) to denote the number of edges in the unique path between *u* and *v* in *T*. Any edge $(u, v)\in E(T)$ induces a two-part partition $P(e)=\{P_u, P_v\}$ of *L*, where $P_u=\{\ell (x) \,|\; d(x, u)<d(x, v)\}$, which contains *u*, and $P_v=\{\ell (y) \;|\; d(y, v)< d(y, u)\}$, which contains *v*. Let us define ${{\mathcal {P}}}(T)=\{P(e)\;|\; e\in E(T)\}$.

#### Proposition 3

For any two 1-labeled trees *S*, *T* of $G_{\mathrm {gnni}}(L)$,

$$\begin{aligned} \frac{1}{2} |{{\mathcal {P}}}(S) \Delta {{\mathcal {P}}}(T)| \le d_{\mathrm {gnni}}(S, T) < |{{\mathcal {P}}}(S) \Delta {{\mathcal {P}}}(T)|, \end{aligned}$$

where $\Delta$ is the set symmetric difference operator.

#### Proof

Let *S* and *T* be two trees with *n* nodes over the same label set. The first inequality is derived from the following two facts:

- ${{\mathcal {P}}}(S)\setminus {{\mathcal {P}}}(T)$ contains exactly one partition *P*(*e*) if *T* is obtained from *S* by applying a gNNI on *e* for each $e \in E(S)$;
- $A\Delta B \subseteq (A\Delta C) \cup (C\Delta B)$ for any three sets.

Let $d_{\mathrm {gnni}}(S, T)=d$. There are a sequence of 1-labeled trees

$$\begin{aligned} T=T_0, T_1, \cdots , T_{d}=S \end{aligned}$$

such that $T_i$ can be obtained from $T_{i-1}$ by applying a gNNI operation for $i=1, 2, \cdots , d$. Note that only one edge-induced partition of $T_{i-1}$ is not an edge-induced partition in $T_{i}$ and vice versa. Since the $T_i$’s are 1-labeled, we have that $|{{\mathcal {P}}}(T_{i-1})\Delta {{\mathcal {P}}}(T_i)|=2$ for each *i*. Since the $\Delta$ operator satisfies the triangle inequality, we have that

$$\begin{aligned} |{{\mathcal {P}}}(T)\Delta {{\mathcal {P}}}(S)|\le \sum ^{d}_{i=1}|{{\mathcal {P}}}(T_{i-1})\Delta {{\mathcal {P}}}(T_i)|=2d \end{aligned}$$

and thus $$\frac{1}{2}|{{\mathcal {P}}}(T)\Delta {{\mathcal {P}}}(S)|\le d=d_{\mathrm {gnni}}(S, T).$$

To prove the upper bound, we let $m=|{{\mathcal {P}}}(S) \cap {{\mathcal {P}}}(T)|$ and let

$$\begin{aligned} {{\mathcal {P}}}(S) \cap {{\mathcal {P}}} (T)=\, & {} \{P(e'_1), P(e'_2), \cdots P(e'_m)\}\\=\, & {} \{P(e''_1), P(e''_2), \cdots P(e''_m)\}, \end{aligned}$$

where $e'_i\in E(S), e''_i\in E(T)$ such that $P(e'_i)=P(e''_i)$ for each *i*. $S - \{ e'_i | 1\le i\le m\}$ is the disjoint union of $m+1$ subtrees $S_j$ ($0\le j\le m$); similarly, $T-\{e''_i | 1\le i\le m\}$ is the disjoint union of $m+1$ subtrees $T_i$ ($0\le i\le m$). Additionally, for each $0\le j\le m$, a unique index *k*(*j*) exists such that $S_j$ and $T_{k(j)}$ contain the same number (say $o_i$) of nodes, where $o_i\ge 1$. Note that

1 $$\begin{aligned} |{{\mathcal {P}}}(S) \Delta {{\mathcal {P}}}(T)|+2m=|E(S)|+|E(T)|=2n-2. \end{aligned}$$

There are three possible cases for each pair of subtrees $S_j$ and $T_{k(j)}$. First, if $o_j=1$, we do not need to do any local adjustments of $S_j$ to transform *S* to *T*.

If both $S_{j}$ and $T_{k(j)}$ contain two nodes *u* and *v*, (*u*, *v*) is then the only edge of $S_j$ and $T_{k(j)}$. This implies that the two nodes are the ends of different edges of ${{\mathcal {P}}}(S) \cap {{\mathcal {P}}} (T)$ in *S* and *T*, and thus we need one gNNI to switch these two nodes in *S* so that they are incident to the same edges as in *T* after the operation.

If both $S_{j}$ and $T_{k(j)}$ contain $o_j$ ($\ge 3$) nodes, we select an internal node *s* of $S_j$ and a node *t* of $T_{k(j)}$ such that *s* and *t* have the same label. By continuing to apply, at most, $o_j-3$ gNNIs on the edges incident to *s*, we can transform $S_{j}$ into a star tree *C* centered on *s*, as *s* is an internal node. Similarly, by applying $o_j-2$ gNNIs at most, we can transform *C* into $T_{k(j)}$. Taken together, the two transformations give a transformation from $S_{j}$ into $T_{k(j)}$ consisting of at most $2o_j-5$ gNNIs at most.

Let $m_i$ be the number of subtrees $S_j$ such that $|S_j|=i$ for $i=1, 2$ and let $m_3$ be the number of subtrees $S_j$ such that $|S_j|\ge 3$. We have that $m_1+m_2+m_3=m+1$ and there are $n-m_1-2m_2$ nodes in the union of all subtrees $S_j$ in Case 3. By combining all the transformations from $S_j$ to $T_{k(j)}$, we can transform *S* to *T* in *c* gNNIs at most, where:

$$\begin{aligned} c= & {} 0 + m_2+ [2(n-m_1-2m_2) -5m_3]\\= & {} 2n-2m_1-3m_2 -3m_3-2m_3\\= & {} 2n-2m_1-3m_2 -3m_3-2(m+1-m_1-m_2)\\= & {} 2n-2m-2-m_2-3m_3. \end{aligned}$$

Since $m_2\ge 0$ and $m_3\ge 0$, by Eqn. (1), $c\le 2n-2m-2= |{{\mathcal {P}}}(S) \Delta {{\mathcal {P}}}(T)|$.

### The RF distance on 1-labeled trees on the same label set

**Fig. 3:**
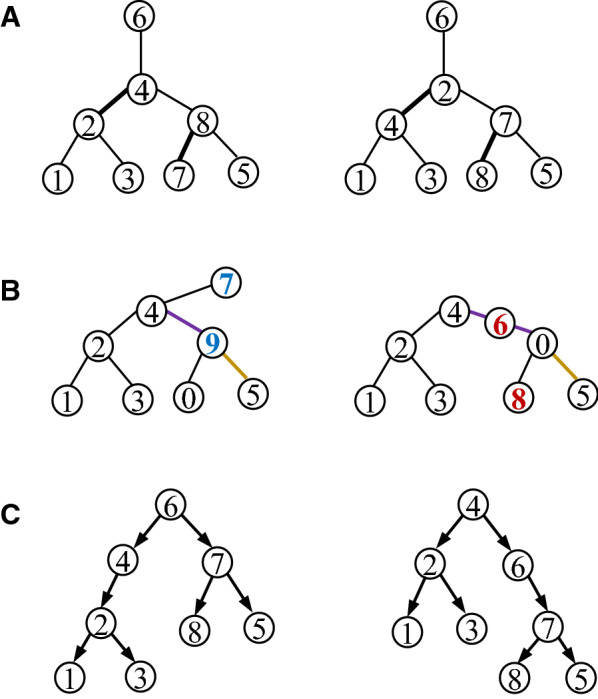
Illustration of the RF distance and the Bourque distance. **A** Two unrooted 1-labeled trees. The RF distance between them is 4, as in the left tree, the edges (2, 4) and (7, 8) induces two partitions that are not found in the right tree and vice versa. **B** The labels 0–5 are the labels appearing in the two trees. The Bourque distance between them is 9 (see the main text for details). **C** The two labeled trees are rooted at different nodes. The RF distance between the left tree and the right tree is 2, as the partitions induced by (6, 4) of Tree A and (4, 6) of Tree B are different

Let *S* and *T* be two 1-labeled trees. $|{{\mathcal {P}}}(S) \Delta {\mathcal P}(T)|$ is called the *RF distance* between *S* and *T*, denoted $\mathrm{RF}(S, T)$ [8]. For example, in the left tree given in Fig. 3A, the edge (2, 4) (bold) induces the two-part partition $\{\{1, 2, 3\}, \{4, 5, 6, 7, 8\}\}$; the edge (7, 8) (bold) induces $\{\{7\}, \{1, 2, 3, 4, 5, 6, 8\}\}$. These two partitions are not equal to any edge-induced partition in the right tree. Similarly, we have that the two-part partitions induced by the edges (2, 4) and (7, 8) in the right tree are not found in the left tree. One can also verify that the other five edge-induced partitions in both trees are identical. Hence, the RF distance between the left and right trees is 4.

Like the phylogenetic tree case, it is easy to see that the RF satisfies the non-negativity, symmetry and triangle inequality conditions.

## Generalizations of the RF distance for labeled trees on different label sets

Let us consider labeled trees of different sizes or whose label sets are not the same. The RF distance between any pair of such trees is simply equal to the total number of edges in the trees and thus fails to capture their dis-similarity. Here, we generalize the RF distance in order to measure the dis-similarity of such pairs of trees better.

### Bourque distances

For a labeled tree *S*, we use ${{\mathcal {L}}}(S)$ to denote the label set of *S*. Since each node of *V*(*S*) is labeled with a non-empty subset of ${{\mathcal {L}}}(S)$, each edge $e=(u, v)$ induces the two-part partition $P(e)=\{L(u), L(v)\}$, where $L(u)=\cup _{x\in V(S): d(x, u)<d(x, v)} \ell (x)$ and $L(v)=\cup _{y\in V(S): d(y, v)<d(y, u)} \ell (y)$.

Let *T* be another labeled tree such that ${{\mathcal {L}}}(S)\cap {{\mathcal {L}}}(T)\ne \emptyset .$ We define $C={\mathcal L}(S)\cap {{\mathcal {L}}}(T)$.

For $e'\in E(S)$, we assume that the two-part partition induced by $e'$ is $P(e')=\{X, {{\mathcal {L}}}(S)\setminus X\}$, where $X\subset {{\mathcal {L}}}(S)$. $P(e')$ is said to be similar to a two-part partition $P=(C', C'')$ of *C* if the following condition is satisfied:

- $$\{X\cap C, ({{\mathcal {L}}}(S)\setminus X)\cap C\}= \{C', C'' \}$$.

We use $\sim$ to denote the similarity relationship.

#### Remarks

(1) The similarity relation is a many-to-many relation in the product space of edge-induced partitions ${{\mathcal {P}}(S)}\times {{\mathcal {P}}}(T)$. (2) If ${{\mathcal {L}}}(S)= {{\mathcal {L}}}(T)$, the similarity becomes the equal relation.

#### Definition 2

Let *S* and *T* be two labeled trees and let ${{\mathcal {P}}}=\{\{C', C\setminus C'\} : \emptyset \ne C'\subset C,\; C'\ne C \}$. The Bourque metric *B*(*S*, *T*) between *S* and *T* is defined as:

2 $$\begin{aligned} |{{\mathcal {P}}}(T)\cup {{\mathcal {P}}}(S)|-\sum _{P\in {{\mathcal {P}}}} \min \left( |{\mathcal Q}'_S(P)|, |{{\mathcal {Q}}}''_T(P)|\right) \end{aligned}$$

where

$$\begin{aligned} {{\mathcal {Q}}}'_S(P)= & {} \{Q' \in {{\mathcal {P}}}(S): Q'\sim P\},\\ {{\mathcal {Q}}}''_T(P)= & {} \{Q''\in {{\mathcal {P}}}(T) : Q''\sim P\}. \end{aligned}$$

The rationale behind the Bourque distance is that we “correct” the RF distance by those partitions, that would be shared between both trees when labels unique to either of the two trees were ignored. For example, in Fig. 3B, the labels $\{7, 9\}$ that appear in the left tree are not found in the right tree, whereas the labels $\{6, 8\}$ that appear in the right tree are not found in the left tree. Therefore, none of the seven edge-induced partitions in either tree is found in the other. This implies that the RF distance between the two trees is 14. Since the labels appearing in both trees are $\{0, 1, 2, 3, 4, 5\}$, the edge (4, 9) (purple) of the left tree induces the same partition, $\{\{1, 2, 3, 4\}, \{0, 5\}\}$ of $\{0, 1, 2, 3, 4, 5\}$ as the edges (4, 6) and (6, 0) (purple) of the right tree. Furthermore, the edge (1, 2) (resp. (2, 3) and (2, 4)) induces the same partition of $\{0, 1, 2, 3, 4, 5\}$ in both trees; and the edge (9, 5) of the left tree induces the same partition of $\{1, 2, 3, 4, 5\}$ as the edge (0, 5) of the right tree. Therefore, the Bourque distance between both trees is $14-5=9$.

#### Proposition 4

Let *S* and *T* be two labeled trees with *s* and *t* edges, respectively.

(i) If $${{\mathcal {L}}}(S)={{\mathcal {L}}}(T)$$, $${B}(S, T)= \mathrm{RF}(S, T)\ge |s- t|$$.
(ii) If $${{\mathcal {L}}}(S)\cap {{\mathcal {L}}}(T)=\emptyset$$, $${B}(S, T)=\mathrm{RF}(S, T)=s+t$$.
(iii) If $${{\mathcal {L}}}(S)\ne {{\mathcal {L}}}(T)$$, $$\max (s, t)\le {B}(S, T)\le s+t$$.

#### Proof

Let *S* and *T* be two labeled trees. (i) Without loss of generality, we assume $s\ge t$. If ${{\mathcal {L}}}(S)={{\mathcal {L}}}(T)$, the first and second term of Eqn.(2) equals $s+t-|{\mathcal P}(S)\cap {{\mathcal {P}}}(T)|$ and $-|{{\mathcal {P}}}(S)\cap {{\mathcal {P}}}(T)|$, respectively. Thus, ${B}(S, T)= \mathrm{RF}(S, T)= s+t-2 |{{\mathcal {P}}}(S) \cap {{\mathcal {P}}}(T)| \ge s+t-2t=s-t$.

(ii) If ${{\mathcal {L}}}(S)\cap {{\mathcal {L}}}(T)=\emptyset$, then, the first term and second term of Eqn.(2) equals $s+t$ and 0, respectively, as $|{{\mathcal {P}}}(S)\cap {{\mathcal {P}}}(T)|=\emptyset$.

(iii) If ${{\mathcal {L}}}(S)\ne {{\mathcal {L}}}(T)$, $|{{\mathcal {P}}}(T)\cup {\mathcal P}(S)|=s+t$, imply that $B(S, T)\le s+t$. Moreover, by definition, we have:

$$\begin{aligned}&\sum _{P\in {{\mathcal {P}}}}\min \left( |{{\mathcal {Q}}}'(P)|, |{{\mathcal {Q}}}''(P)|\right) \\\le & {} \min \left( \sum _{P\in {{\mathcal {P}}}}|{{\mathcal {Q}}}'_S(P)|, \sum _{P\in {{\mathcal {P}}}}|{{\mathcal {Q}}}''_T(P)|\right) \\\le & {} \min (|{{\mathcal {P}}}(S)|, |{{\mathcal {P}}}(T)|)= \min (s, t) \end{aligned}$$

and:

$$\begin{aligned} B(S, T)\ge s+t - \min (s, t)=\max (s, t). \end{aligned}$$

Additionally, we also have the following fact, which is proved in Additional file 1.

#### Proposition 5

The Bourque metric is a distance metric in the space of labeled trees; in other words, it satisfies the non-negativity, symmetry and triangle inequality conditions.

#### Proposition 6

The Bourque distance between two labeled trees *S* and *T* can be computed in linear time $O(|{{\mathcal {L}}}(S)|+{{\mathcal {L}}}(T)|$.

#### Proof

The proof is an adaption of the proof by Day for computing the Robinson–Foulds distance of rooted leaf-labelled trees in linear time [38]. We assume node labels are integers (otherwise, we apply hashing to convert the labels into integers). By indexing labels with integers and filling a hash table, we can determine the set *C* of node labels that are in both trees. If *C* is empty, we have $B(S,T) = s+t$. Otherwise, we remove all labels that are not in *C* from the two trees *S* and *T*. This may create some nodes *v* with no labels, i. e. $\ell (v) = \emptyset$. We remove leafs with no labels from *S* and the corresponding edges as they do not induce any non-trivial partitions. We then select an arbitrary node *r* that is labeled with at least one label, root *S* at *r* and map the labels to $[ 1, 2, \cdots , |C|]$ based on a pre-order depth-first traversal of *S*. Since node labels occur only once per tree, this mapping is well-defined, and we obtain a new node labelling $\ell '$ for which the elements of each $\ell '(v)$ are consecutive integers and smaller than the elements of $\ell '(w)$ for every *w* accessed after *v* in the pre-order depth-first traversal of *S*. In particular, 1 is a label of the root; for every subtree of the rooted *S*, the union of node labels of all nodes in the subtree is now a consecutive interval. Using efficient data structures, the above tree manipulations amortise to linear time with regard to $|{{\mathcal {L}}}(S)|$. Using a post-order depth-first traversal of the rooted tree *S*, we can obtain all consecutive intervals in linear time with regard to $|{{\mathcal {L}}}(S)|$. Due to nodes with no labels, the same interval can occur multiple times. Therefore we track the counts of the intervals. This can be done efficiently with a hash table.

Now, we relabel the nodes of *T* using the mapping obtained from the pre-order traversal of *S* and root *T* at the node containing the label 1. We perform a post-order depth-first traversal of *T* and obtain the intervals defined by the smallest and largest label of each subtree. In addition we also keep track of the total number of labels in the subtree. If the length of the interval matches this number, the interval is consecutive and thereby the incoming edge to the subtree defines a partition that is also induced by an edge in *S*. The necessary operations amortise to linear time with regard to $|{{\mathcal {L}}}(S)| + |{{\mathcal {L}}}(T)|$ . Since the label 1 is located at the root in both *S* and *T*, the obtained intervals for *S* and *T* are always the part of the partition that does not contain the label 1. Therefore it is sufficient to consider these intervals to compare partitions. Let $Z_S$ be the multi-set of intervals obtained from *S* and $Z_T$ be the multi-set of (consecutive) intervals obtained from *T*, then we obtain the second part of Eq. (2) by summing over the smaller prevalence of each interval in either *T* or *S*. This can be accomplished in linear time using two hash tables to track the prevalence of the intervals in each tree. The first part of Eq. (2) is just the number of edges in *S* and *T* in the case ${{\mathcal {L}}}(S)\ne {{\mathcal {L}}}(T)$. Pseudocode of the algorithm is given in Section 4 of Additional file 1. In case ${{\mathcal {L}}}(S) = {{\mathcal {L}}}(T)$, *B*(*S*, *T*) is simply the size of the intersection of $Z_S$ and $Z_T$. This concludes that the Bourque distance can be computed in linear time.

### High-order Bourque distances for labelled trees

Like the RF distance, the Bourque distance has the tendency to overpenalize certain labeling differences and can saturate quickly (see our validation tests on random trees presented later). In this subsection, we will use the Bourque distances between local subtrees and a matching algorithm ([15, 17, 18]) to define new distance metrics. The new metrics will take more values than the basic version.

Let *T* be a labeled tree and $u\in V(T)$. For an integer $k>0$, the *k*-star subtree $C_k(u)$ centered at *u* is defined as the subtree induced by the vertex set $\{v\in V(T) : d(u, v)\le k\}$ in *T*. For any pair of labeled trees *S* and *T* of *n* and $n'$ nodes, respectively, such that $n \le n'$, define $\mathrm {BG}_k(S, T)$ to be the complete weighted bipartite graph with two node parts $\{\emptyset _1, \cdots \emptyset _{n'-n}\}\cup V(S)$ and *V*(*T*), where each $\emptyset _i$ is just a copy of the empty graph; the Bourque distance $B(C_k(x), C_k(y))$ is assigned to the edge (*x*, *y*) as a weight for every $x\in V(S)$ and $y\in V(T)$ and a weight of $|E(C_{k}(y))|$ is assigned to the edge $(\emptyset _i, y)$ for any $\emptyset _i$ and $y\in V(T)$. Although $C_k(x)$ can be identical for different nodes *x*, $\mathrm {BG}_k(S, T)$ always has $2n'$ nodes.

#### Definition 3

Let *S* and *T* be two labeled trees and $k \ge 1$. The *k*-Bourque distance $B_k(S, T)$ is defined to be the minimum weight of a perfect matching in $\mathrm {BG}_k(S, T)$.

#### Proposition 7

The *k*-Bourque distances have the following properties:

1. For any 1-labeled trees *S* and *T* such that $|V(S)|= |V(T)|=n$, $B_k(S, T)=n \cdot {B}(S, T)$ for any $k\ge \max (\mathrm{diam}(S), \mathrm{diam} (T))$, where $\mathrm{diam}(X)$ is the diameter of *X* for $X=S, T$.
2. $B_k(S, T)$ satisfies the non-negativity, symmetry and triangle inequality conditions for each $k\ge 1$.

#### Proof

The full proof appears in the Additional file 1.

#### Remark

The run time of computing the *k*-Bourque distance for two labeled trees *S* and *T* with *n* and $n'$ nodes, respectively, is $O(\max (n', n)^3)$, as computing the Bourque distances between the *k*-star trees centered at tree nodes takes $O(\max (n', n))$ in the worst case and computing the minimum weight perfect matching in $\mathrm{BG}_{k}(S, T)$ takes $O(\max (n', n)^3)$ time.

## The Bourque distances for mutation trees

In this section, we will describe how to generalize the gNNI and Bourque distances to rooted labeled trees.

### The gNNI for mutation trees

To transform a binary rooted phylogenetic tree into another, we need the so-called rotation operation that allows two nodes *u* and *v* that are connected by an edge to interchange not only one of their children but also their positions (right, Fig. 1B) [40]. A gNNI on a directed edge (*a*, *b*) of a rooted tree rewires some outgoing edges from *a* to *b* and vice versa and/or rewires the incoming edges to both *a* and *b* simultaneously (right, Fig. 2B). More precisely, the gNNI is defined on rooted labeled trees as follows:

#### Definition 4

Let *T* be a rooted labeled tree and $e=(a, b)\in E(T)$ (where *b* is a child of *a*). An NNI operation on *e* transforms *T* by selecting a subset of edges $C_a=\{(a, x)\}$ that leave *a*, where $(a, b)\not \in C_a$, and a subset of edges $C_b=\{(b, y)\}$ that leave *b* and then either (i) replacing each edge (*a*, *x*) of $C_a$ with (*b*, *x*) and each edge (*b*, *y*) of $C_b$ with (*a*, *y*) (left, Fig. 2B) or (ii) rewiring the edges in $C_a$ and $C_b$ as in (i) as well as replacing the unique edge (*z*, *a*) that enters *a* and (*a*, *b*) with (*z*, *b*) and (*b*, *a*), respectively (right, Fig. 2B).

It is easy to see that for any pair of arbitrary labeled trees *S* and *T*, *S* can be transformed into *T* through a series of gNNIs as long as the labels appearing in the two trees are the same.

### The RF and Bourque distances for mutation trees

In a rooted labeled tree, each directed edge also induces a 2-part partition on the label set. Therefore, the RF distance is well defined even for rooted trees that may not be uniquely labeled.

Let *T* be a rooted labeled tree. Recall that ${{\mathcal {L}}}(T)$ denotes the set of labels appearing in *T*. For a non-root node $u\in V(T)$, we use $L_T(u)$ to denote the set of the labels of *u* and its descendants, i.e.

3 $$\begin{aligned} L_T(u)=\cup _{x\in \{u\}\cup D_T(u)} \ell (x). \end{aligned}$$

The unique edge entering *u* induces then an “ordered” two-part partition $(L_T(u), {{\mathcal {L}}}(T) \setminus L_T(u))$, which is an ordered pair of the two complementary subsets of ${{\mathcal {L}}}(T)$. Since the root of a rooted tree is a distinct node of the tree, we assume that the root is contained in the second part of an edge-induced partition. Hence, two edge-induced ordered partitions $P'$ and $P''$ are *equal* if and only if the first part of $P'$ is equal to the first component of $P''$ and the second part of $P'$ is equal to the second component of $P''$. This is particularly useful when comparing two rooted trees with different roots. Let us define ${\mathcal OP}(T)$ to be the set of all edge-induced ordered partitions of *T*.

#### Definition 5

For two rooted labeled trees *S* and *T*, the RF distance $\mathrm{RF}(S, T)$ between *S* and *T* is defined as $|{\mathcal OP}(T)\;\Delta \;{\mathcal OP}(T)|$.

For example, the two trees given in Fig. 3C are obtained from rooting a unrooted labeled tree at different nodes. Only the partition induced by the edge (6, 4) of the left tree is not found in the right tree and vice versa. Hence, the RF distance between these two trees is 2.

#### Proposition 8

Let *S* and *T* be two rooted labeled trees of equal size that have the same labels.

1. Let $t\in V(T)$ such that it has the same label as the root $r_S$ of *S* and let $r_T$ be the root of *T*. We have that $RF(S, T) \ge 2 d_{T}(r_T, t)$, where $d(r_T, t)$ is the distance between $r_T$ and *t* in *T*.
2. $$\frac{1}{2}RF(S, T)\le d_{\mathrm {gnni}}(S, T) \le RF(S, T)$$.

#### Proof

(1). Let the path between $r_T$ and *t* be $r_T=t_0, t_1, t_2, \cdots , t_d=t$, where $d=d_T(r_T, t)$. All label sets $L_{T}(t_i)$ contain the label $\ell (r_S)$. However, only $L_T(t_0)$ is an element of $\{ L_S(u)\;|\; u\in V(S)\}$. Furthermore, since both trees have the same number of nodes and edges, at least *d* subsets of $\{ L_S(u)\;|\; u\in V(S)\}$ are not found in $\{L_T(v)\;|\; v\in V(T)\}$. Hence, $RF(S, T)\ge 2d$.

(2) The proof is similar to that of Proposition 3.

Similarly, we can generalize the similarity relationship of edge-induced partitions. For two non-root nodes $u\in V(S)$ and $v\in V(T)$, the ordered partitioned induced by the edges entering *u* and *v* are *similar* if and only if

$$\begin{aligned} (L_S(u)\cap C, [{{\mathcal {L}}}(S)\setminus L_S(u)]\cap C)= (L_T(v)\cap C, [{{\mathcal {L}}}(T)\setminus L_T(v)]\cap C) \end{aligned}$$

and $\emptyset \ne L_S(u)\cap C\ne C$, where $C={{\mathcal {L}}}(S)\cap {\mathcal L}(T)$, denoted by

$$\begin{aligned} (L_S(u), {{\mathcal {L}}}(S)\setminus L_S(u)) \sim (L_T(v), {{\mathcal {L}}}(T)\setminus L_T(v)). \end{aligned}$$

#### Definition 6

The Bourque distance *B*(*S*, *T*) between two rooted labeled trees *S* and *T* is defined to be:

4 $$\begin{aligned} |{\mathcal OP}(S)\cup {\mathcal OP}(T)|- \sum _{P\in {{\mathcal {P}}}} \min (|{\mathcal O}'_S(P)| , |{{\mathcal {O}}}''_T(P)|), \end{aligned}$$

where

$$\begin{aligned} {{\mathcal {O}}}'_S(P)=\{P'\in {\mathcal OP}(S) \;:\; P'\sim P\},\\ {{\mathcal {O}}}''_T(P)=\{P''\in {\mathcal OP}(T) \;:\; P''\sim P\}. \end{aligned}$$

#### Proposition 9

The Bourque distance between two mutation trees *S* and *T* can be computed in linear time $O(|{{\mathcal {L}}}(S)|+{\mathcal L}(T)|$.

The proof of Proposition 9 is analogous to Proposition 6, but instead of rooting the tree at a random node, we use the actual root as start for the tree traversal. By construction, the generated intervals will not contain any root labels and thus represent the left component of the partition. Hence, partitions can still be counted and compared based on the intervals obtained from the tree traversals as detailed in the proof of Proposition 6. Therefore the Bourque distance can be computed in linear time also for mutation trees.

### High-order Bourque distances

Let *S* and *T* be two rooted labeled trees and $k\ge 1$. Recall that $D_{T}(u)$ denotes the set of descendants of *u* in *T*. Define further $D^{(k)}_{T}(u)=\{ v \in D_T(u) : d_T(u, v)\le k\}$ and $T^{(k)}(u)$ to be the subtree of *T* induced by $D^{(k)}_{T}(u)$.

Like the unrooted tree case, we define the *k*-Bourque distance $B_k(S, T)$ to be the minimum weight of a perfect matching in the complete weighted bipartite graph $G_k(S, T)$. Here, assuming $n=|V(S)|\le |V(T)|=n'$, $G_k(S, T)$ has the vertex set:

$$\begin{aligned} \{\emptyset _i, x : 1\le i\le n'-n; \; x\in V(S)\} \cup \{y : y\in V(T)\} \end{aligned}$$

and the edge set:

$$\begin{aligned} \{\emptyset _i, x : 1\le i\le n'-n; x\in V(S)\}\times \{y : y\in V(T)\}, \end{aligned}$$

together with the following edge-weight function *w*:

$$\begin{aligned} w((x, y))= B(S^{(k)}(x), T^{(k)}(y)), \\ w((\emptyset _i, y))= |E(T^{(k)}(y))|. \end{aligned}$$

where each $\emptyset _i$ is a copy of the empty graph.

**Fig. 4:**
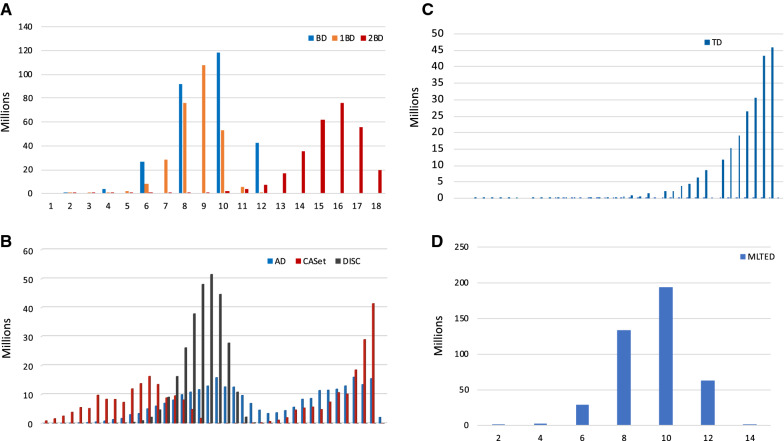
The frequency distributions of all possible pairwise distances in the space of rooted 1-labeled trees with seven nodes. **A** The distributions for the BD, 1-BD and 2-BD metrics.**B** The distributions for the AD, CASet and DISC measures. Here, all the pairwise distances were binned into 40 equal intervals $\left( \frac{i}{40}, \frac{i+1}{40}\right]$, $0\le i\le 39$. **C** The distribution for the TD measure. **D** The distribution for the MLTED measure. *BD* Bourque distance, *AD* ancestor distance, *CASet* common ancestor set distance, *DISC* distinctly inherited set, *TD* triplet-based distance, *MLTED* multi-label tree edit distance

## Comparison of eight distance measures on rooted labeled trees

In this section, we compare the Bourque distance (BD) against the 1-Bourque distance (1-BD), the 2-Bourque distance (2-BD) and five previously published distance measures: Common Ancestor Set (CASet) [35], Distinctly Inherited Set Comparison (DISC) [35], an Ancestor Difference measure (AD) [36], a Triplet-based Distance (TD) [34] and the Multi-Labeled Tree Edit Distance (MLTED) measure [37]. A detailed description of these measures is given in Section 3 of the Supplementary file. The gNNI distance is not included in the comparison, as no efficient method for computing it is known.

### Frequency distributions of the pair-wise distances in different metrics

There are 16,807 unrooted and $7\times 16,807$ rooted 1-labeled trees with seven nodes. Let *R* denote the set of such trees and let $R_i$ denote the set of those rooted at Node *i*, where $1\le i\le 7$. Let *d* be a distance function of rooted labeled trees. Clearly, for any *i*, $\{d(x, y): x\in R_i, y\in R_i\}=\{d(x, y) : x\in R_1, y\in R_1\}$; for different nodes *i* and *j*, $\{d(x, y): x\in R_i, y\in R_j\}=\{d(x, y) : x\in R_1, y\in R_2\}$. Therefore, for each measure, we computed the pairwise distances between any $x\in R_1$ and any $y\in R_1\cup R_2$ such that $x\ne y$.

The frequency distributions of the BD, 1-BD and 2-BD are given in Fig. 4A, showing a Poisson distribution as the RF in the unrooted case [41].

The pairwise distances of AD, CASet, DISC and TD range from 0 to 1. Because of over 512 million of pair-wise distances, we binned them into 40 intervals $\left( \frac{i}{40}, \frac{i+1}{40}\right)$, $0\le i\le 39$. The histograms for the frequency distributions of the pairwise distance values for the three measures are given in Fig. 4B. The AD and CASet measures have a similar distribution (blue and red in Fig. 4B), each having two peaks. The pairwise distances between trees rooted at the same node form the first peak, whereas the pairwise distances between trees rooted at different nodes form the second peak. These facts show that AD and CASet are sensitive to the root node. The frequency distribution (black) of the DISC measure appears to be again a kind of Poisson distribution. Whether the pairwise distances of the DISC, 1-BD and 2-BD between all 1-labeled trees with a given number of nodes follow a Poisson distribution or not needs further mathematical investigation. The key point is that the DISC measure and the Bourque metrics have different distributions of pairwise distances from the AD and DISC measures.

The frequency distribution of the TD is clearly different from the AD, CASet and DISC (Fig. 4C). More than 60% of the pairwise distances are greater than 0.9. For the discrete MLTED measure, we observe a Poisson-like distribution similar to the BD metric.

Lastly, for each of the AD, CASet and TD measures, there are many pairs of trees with the same distance value, that have distinct distances in the BD metric. Additional file 2: Fig. S1 give an example for each of these measures.

### Pairwise distances between random trees

We compared the BD, 1-BD, 2-BD, AD, CASet, DISC, TD and MLTED measures on rooted 1-labeled, 30-node trees that were randomly generated as follows. The tree generator first generated a random unrooted 1-labeled 30-node tree $T_0$ and then generated 20,000 random unrooted 1-labeled, 30-node trees in 400 iterations. In the *i*-th iteration, a tree generated in the $(i-1)$-th iteration was randomly selected; five random trees were then generated from the selected tree by applying a random NNI on an edge $e=(u,v)$ that was randomly selected, where *u* was an internal node. Here, a NNI just switched one subtree from the *u* side to *v* and one subtree from the *v* side to *u* if *v* was not a leaf and just moved a subtree from *u* to *v* if *v* was a leaf.

The generated random trees are unrooted 1-labeled trees on $\{0, 1, \cdots , 29\}$. We rooted all the trees at Node 0. To generate random tree with different label sets and/or with multiple-labeled nodes, we first removed three nodes (27, 28, 29), two nodes (28, 29) or one node (29) with probability $\frac{1}{200}$, $\frac{1}{100}$, $\frac{1}{100}$, respectively, in each random tree; we then decided to merge three/two nodes that are not equal Node 0 into one node with multiple labels with probability $\frac{1}{150}$ and $\frac{2}{150}$, respectively. Here, nodes were removed from a tree one by one. When a node was removed, a neighbor of it was randomly selected and the other neighbors were reconnected to the selected one. When it was decided to merge *t* nodes in a tree, then *t* non-0 nodes $u_i$ ($1\le i\le t$) were randomly selected; $u_2, \cdots , u_t$ were removed from the tree and $u_1$ was relabeled with the subset $\{u_1, u_2, \dots , u_t\}$.

**Fig. 5:**
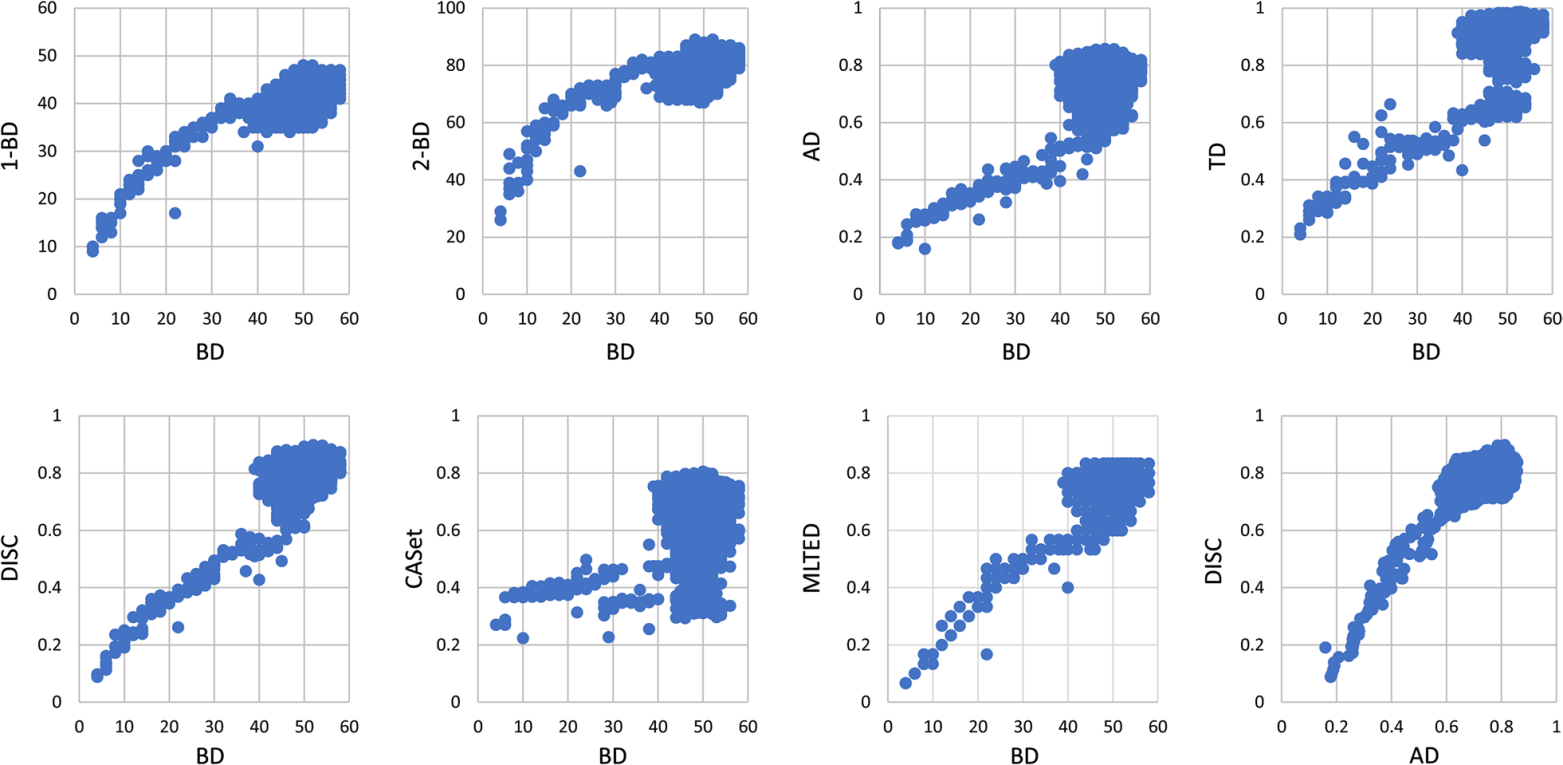
The scatter plots of the Bourque vs the other distance measures between a rooted 1-labeled tree and 19,999 random trees with different label sets or multi-labeled nodes rooted at the same node. *BD* Bourque distance, *AD* ancestor distance, *CASet* common ancestor set distance, *DISC* distinctly inherited set, *MLTED* multi-label tree edit distance, *TD* triplet-based distance

We computed the eight different distance values between $T_0$ and the rest of 19,999, which are summarized in Fig. 5. This produced two interesting findings. First, the BD distances from $T_0$ to the random trees range from 0 to 58; the BD, correlated with 1-BD and 2-BD well with Pearson correlation coefficients (PCC) of 0.5769 and 0.4882, respectively. In particular, when the Bourque distances ranged from 0 to 35, the PCC between BD and 1-BD (resp. 2-BD) is 0.92 (resp. 0.858) (top left panel, Fig. 5). Second, AD, DISC, MLTED and TD correlated with BD (and hence 1-BD and 2-BD) surprisingly well with Pearson correlation coefficients (PCC) from 0.38 to 0.543 even though they are defined differently. However, CASet and BD poorly correlated (middle panel, second row) with PCC 0.103. Third, AS and DISC correlated well with PPC of 0.615.

The same analyses were also done on another dataset which was generated with higher removal and label-merging probability. The probabilities that one, two and three are removed were set to 2/100, 2/100 and 1/100; the probabilities that three and two labels are merged were set to 2/100 and 4/100, The analyses show the same correlation patterns but with lower PCCs (Additional file 3: Figure S2). More precisely, BD correlated with 1-BD, 2-BD, DISC, CASet, AD, TD and MLTED with PCCs 0.368, 0.316, 0.522, 0.108, 0.391, 0.545 and 0.498, respectively.

## Applications to mutation trees

### The distances between three leukemia mutation trees

**Fig. 6:**
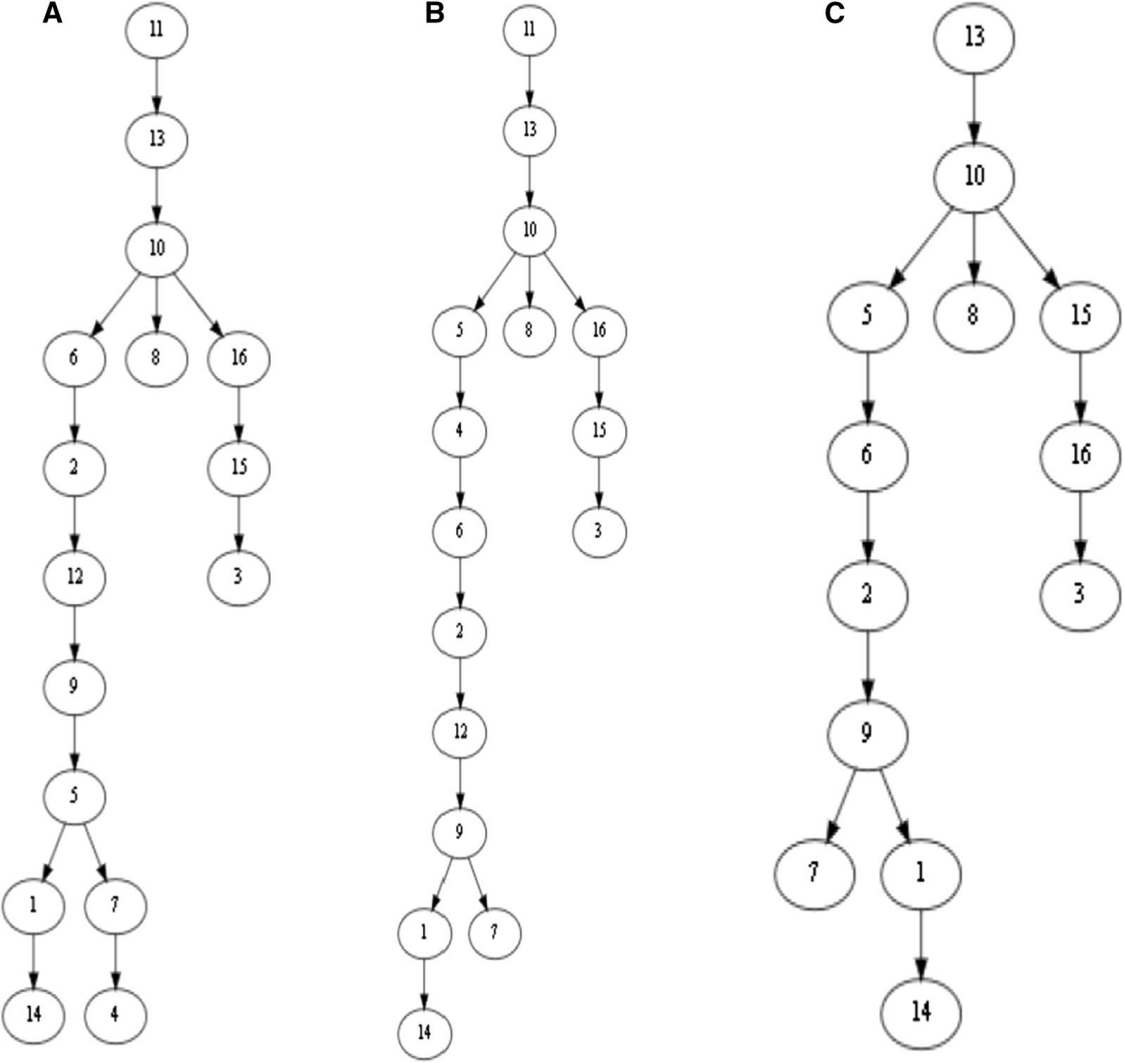
The mutation trees inferred by three different methods for Patient 2 with childhood acute lymphoblastic leukemia that was reported in [20]. **A** The tree inferred by SCITE [28], **B** The tree inferred by B-SCITE [31]. **C** The tree inferred by PhISCS [30]. The mutation trees contain 16 mutated genes: *ATRNL1* (1), *BDNF_AS* (2), *BRD7P3* (3), *CMTM8* (4), *FAM105A* (5), *FGD4* (6), *INHA* (7), *LINXC00052* (8), *PCDH7* (9), *PLEC* (10), *RIMS2* (11), *RRP8* (12), *SIGLEC10* (13), *TRRAP* (14), *XPO7* (15), *ZC3H3* (16)

Single-cell sequencing data are prone to errors. Mutation trees inferred by different methods from the single-cell sequencing data of a patient are often different in both topology and labels, which are mutated genes. Figure 6 shows mutation trees inferred by SCITE [28], B-SCITE [31] and PhISCS [30] for Patient 2, who had childhood acute lymphoblastic leukemia, reported in [20]. Both the SCITE and B-SCITE trees (i.e. Tree A and Tree B) contain 16 mutations, whereas the PhISCS tree (i.e. Tree C) contains only 13 of the 16 mutations.

The pairwise distances between the trees were calculated using the eight distance measures (Table 1). The difference between Tree A and Tree B is mainly the positions of Mutation 4 and Mutation 5 in the long chain on the left. They have the smallest pairwise distance among the three trees for each of the eight measures. Tree B and Tree C have the same topology and are different only in that Mutations 4, 11 and 12 are missing in the latter. For each measure, the distance between Tree B and Tree C is smaller than or nearly equal to the distance between Tree A and Tree C, consistent with intuition.

### Distances between four simulated mutation trees

**Fig. 7:**
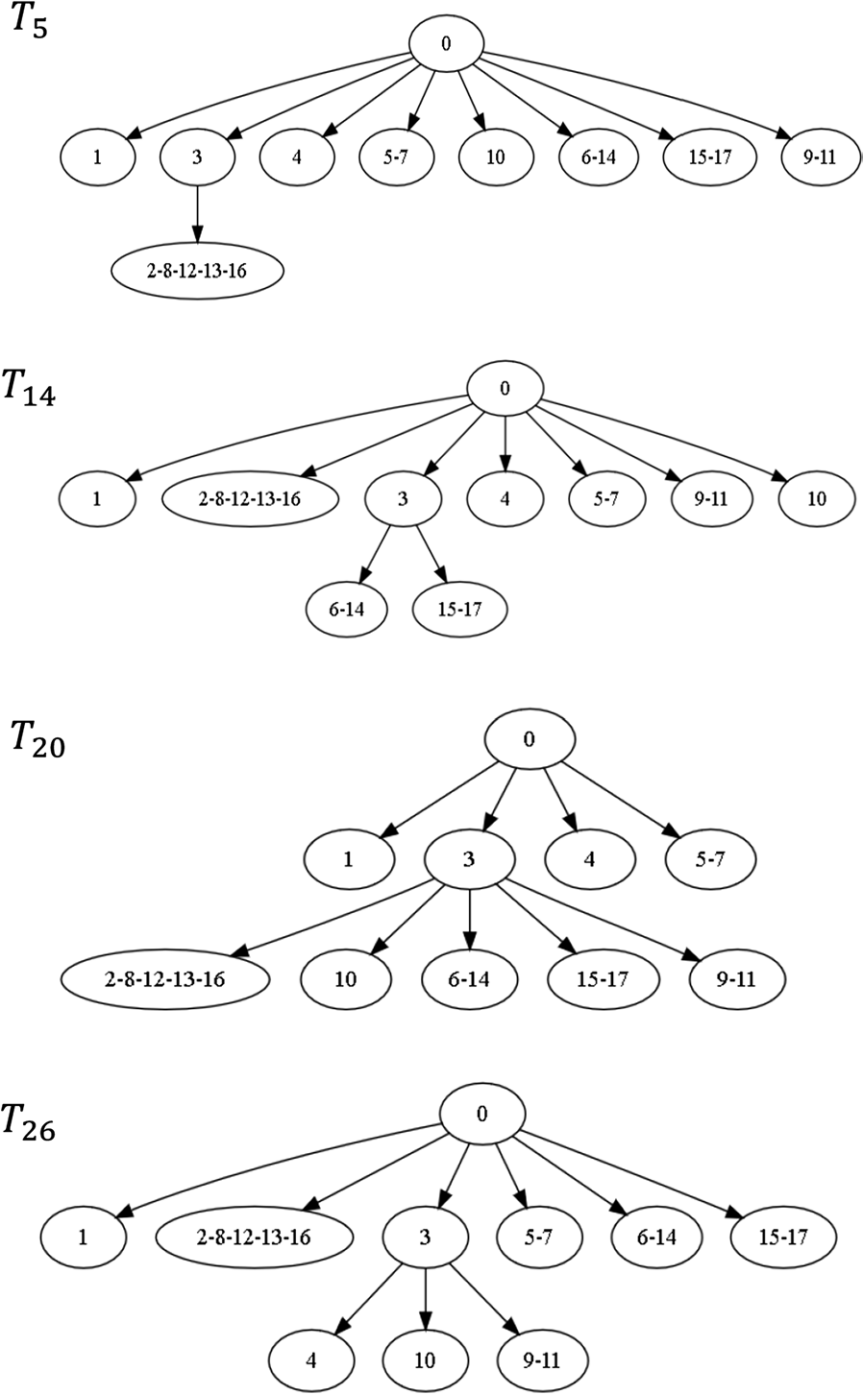
Four simulated mutation trees $T_5, T_{14}, T_{20}$ and $T_{26}$ from the OncoLib database [43]

Figure 7 presents four simulated mutation trees downloaded from the OncoLib database for which the CASet and DISC disagreed significantly [35]. The pairwise distances between the four trees are given in Table 2. Note that the CASet and DISC distances between $T_{5}$ and $T_{20}$ and between $T_{14}$ and $T_{26}$ are different from those reported in [35]. This is because a mutation appearing in a tree node is not an ancestor of another mutation in the same node in our distance calculation. Regardless of the differences between the definitions, our distance computing also shows the disagreement between the CASet and DISC distances. For example, the CASet distance between $T_5$ and $T_{20}$ is four times as large as the CASet distance between $T_{14}$ and $T_{26}$, whereas the DISC distance between the former is smaller than the DISC distance between the latter. This disagreement is also observed on the tree pairs $\{T_5, T_{14}\}$ and $\{T_{20}, T_{26}\}$.

**Table 1:** Pairwise distances between three mutation trees A, B, and C in Fig. 6 in different distance measures

	A & B	A & C	B & C
BD	12	19	16
1-BD	10	18	15
2-BD	27	36	32
MLTED	4	7	5
CASet	0.1079	0.5495	0.5302
DISC	0.2394	0.4331	0.3436
AD	0.1699	0.4874	0.4651
TD	0.2536	0.6393	0.5821

The union extension of CASet and DISC were used to measure the difference between Tree A (or Tree B) and Tree C [35]

Since these four different trees have only one internal edge, the Bourque distance between any two of them is 2. The pairwise 1-BD distances are not much different. However, their differences are reflected in the pairwise 2-BD distances.

**Table 2:** Pairwise distances between trees in Fig. 6 in different distance measures

	$T_5$ & $T_{14}$	$T_5$ & $T_{20}$	$T_5$ & $T_{26}$
BD	2	2	2
1-BD	9	10	10
2-BD	12	14	13
MLTED	6	10	6
CASet	0.0523	0.1830	0.0523
DISC	0.3807	0.2402	0.3807
AD	0.2500	0.1944	0.2500
TD	0.1961	0.4363	0.2120
	$T_{14}$ & $T_{20}$	$T_{14}$ & $T_{26}$	$T_{20}$ & $T_{26}$
BD	2	2	2
1-BD	9	9	10
2-BD	13	12	13
MLTED	14	10	12
CASet	0.1961	0.0392	0.2157
DISC	0.2483	0.3529	0.3039
AD	0.2222	0.2222	0.2778
TD	0.4669	0.2659	0.4951

## Conclusions

We have introduced the Bourque and *k*-Bourque metrics for both unrooted labeled trees and mutation trees. These distances are natural generalizations of the RF distance (see Definitions 2 and 6). We demonstrate, through a simulation, that they correlate with the CASet, DISC and AD distance measures for similar trees, but have different distributions of pairwise distances between all 1-labeled trees with a fixed number of nodes. The advantages of the Bourque metric over CASet and DISC include that it is a distance metric and computable in linear time (Table 3). The *k*-Bourque metrics refine the Bourque metric.

**Table 3:** Summary of the features of different distance measures

	Time complexity	Is metric?	Tree space
gNNI	?	Yes	Unrooted, rooted
BD	Linear	Yes	Unrooted, rooted
*k*-BD	Cubic	Yes	Unrooted, rooted
TD	Cubic [34]	No	Rooted
CASet	Cubic [35]	No	Rooted
DISC	Cubic [35]	No	Rooted
AD	Linear [36]	No	Rooted
MLTED	Polynomial [37]	No	Rooted

Another contribution is a new connection between the RF and gNNI metrics on labeled trees. A few theoretical questions arise from the connection. Is computing the gNNI distance for labeled trees NP-complete? What is the maximum value of the NNI distance between two binary 1-labeled trees? Can the RF distance be used to define a polynomial time algorithm with approximation ratio $<2$ for the gNNi distance?

General mathematical questions also arise from the development of new metrics for comparisons of mutation trees. One is investigating mathematical relationships between the proposed metrics. Another is determining the distributions of pairwise distances between all the 1-labeled trees of the same size. For example, is the distribution Poisson for the Bourque metrics?

Finally, further generalisations of the Bourque distance will be interesting to study in the future, in particular for mutation trees where labels may occur multiple times in different nodes [34]. The motivation for this generalisation comes from the observation that in tumours the same mutations can happen independently in multiple subclones and can also be lost again over time [42].

## Supplementary Information

**Additional file 1.** Analysis of DEGs in the two hemispheres of the mPFC in mice with social defeat stress versus non-stressed mice. Significant DEGs with a FDR adjusted *p*-value cutoff of 0.05 are shown. AveExpr, averaged expression of microarray genes; t, moderated t-statistic; B, B-statistic.

**Additional file 2: Figure S1.** Let T_0_ be the rooted star tree whose root is 1 and whose leaves are 2 to 7. A. Two rooted trees such that the ancestor difference measure between T_0_ and them are 0.1428, but the Bourque distance between T_0_ and them are 2 and 4. B. Two rooted trees such that the triplet-based distances between T_0_ and them are 0.3715, the common ancestor set measure between T_0_ and them are 0.0238, but the Bourque distance between T_0_ and them are 2 and 4.

**Additional file 3: Figure S2.** The scatter plots of the Bourque distance vs the other distance measures between a rooted 1-labeled tree and 19,999 random trees with different label sets or multi-labelled nodesrooted at the same node in the second dataset generated with higher probability that are given inthe main text. BD: Bourque distance; AD: Ancestor distance; CASet: Common Ancestor Set distance;DISC: Distinctly Inherited Set; MLTED: Multi-label tree edit distance; TD: Triplet-based distance.

## Data Availability

Data sharing is not applicable to this article as no biological datasets were generated or analysed during the study. The computer programs used for the study can be downloaded from https://github.com/LX-Zhang/Bourque_Distances
