# Supplementary material for: The Bourque distances for mutation trees of cancers
Source: Algorithms Mol Biol. 2021 Jun 10;16:9. doi: 10.1186/s13015-021-00188-3 (PMC8193869; doi:10.1186/s13015-021-00188-3)
Supplement: Supplementary file 1 — Additional file 1. Analysis of DEGs in the two hemispheres of the mPFC in mice with social defeat stress versus non-stressed mice. Significant DEGs with a FDR adjusted p-value cutoff of 0.05 are shown. AveExpr, averaged expression of microarray genes; t, moderated t-statistic; B, B-statistic. [file 13015_2021_188_MOESM1_ESM.pdf]

Additional Document
for
The Bourque Distances for Mutation Trees of Cancers

K. Jahn, N. Beerenwinkel, LX Zhang

Jan. 2021

1 Proof of Proposition 5

Proposition 5 *The Bourque metric is a distance metric; in other words, it satisfies the non-negativity, symmetry and the triangle inequality conditions.*

Proof. The non-negativity follows Proposition 4. The symmetric property of the Bourque metric follows from its definition. We now prove the triangle inequality.

Let T_1, T_2 and T_3 be three labeled trees. We consider the following three cases to prove $B(T_1, T_2) \leq B(T_1, T_3) + B(T_3, T_2)$.

Case 1. $\mathcal{L}(T_1) = \mathcal{L}(T_3) = \mathcal{L}(T_2)$. In this case, $B(T_i, T_j) = \mathrm{RF}(T_i, T_j)$. The triangle inequality for these three trees follows from the fact that the RF distance satisfies the triangle inequality.

Case 2. $\mathcal{L}(T_1) \neq \mathcal{L}(T_3)$ and $\mathcal{L}(T_3) \neq \mathcal{L}(T_2)$.

Since $\mathcal{P}(T_1) \cap \mathcal{P}(T_2) = \emptyset$, $B(T_1, T_2) \leq |\mathcal{P}(T_1) \cup \mathcal{P}(T_2)| = |\mathcal{P}(T_1)| + |\mathcal{P}(T_2)|$. By the part iii of Proposition 4, $\mathcal{L}(T_i) \neq \mathcal{L}(T_3)$ implies that

$$B(T_i, T_3) \geq \max\left(|\mathcal{P}(T_i)|, |\mathcal{P}(T_3)|\right), \ \ i = 1, 2.$$

Therefore,

$$\begin{aligned} B(T_1, T_3) + B(T_1, T_3) &\geq \max\left(|\mathcal{P}(T_1)|, |\mathcal{P}(T_3)|\right) + \max\left(|\mathcal{P}(T_2)|, |\mathcal{P}(T_3)|\right) \\ &\geq |\mathcal{P}(T_1)| + |\mathcal{P}(T_2)| \geq B(T_1, T_2). \end{aligned}$$

Case 3. $\mathcal{L}(T_1) = \mathcal{L}(T_3) \neq \mathcal{L}(T_2)$ or $\mathcal{L}(T_1) \neq \mathcal{L}(T_3) = \mathcal{L}(T_2)$.

Note that these conditions are symmetric. Hence, we just need to prove that the triangle inequality holds if the first condition is satisfied.

Let $\mathcal{P}$ be the set of 2-part partitions of $\mathcal{L}(T_1) \cap \mathcal{L}(T_2)$. Since $\mathcal{L}(T_1) = \mathcal{L}(T_3)$,

$$B(T_1, T_3) = |\mathcal{P}(T_1) \Delta \mathcal{P}(T_3)| = |\mathcal{P}(T_1)| + |\mathcal{P}(T_3)| - 2|\mathcal{P}(T_3) \cap \mathcal{P}(T_1)|. \quad (A.0)$$

Since $\mathcal{L}(T_1) \neq \mathcal{L}(T_2)$,

$$
\begin{aligned}
& B(T_1, T_2) \\
= \; & |\mathcal{P}(T_1)| + |\mathcal{P}(T_2)| - \sum_{P \in \mathcal{P}} \min\left(|\mathcal{Q}'_{T_1}(P)|, |\mathcal{Q}''_{T_2}(P)|\right). \qquad (A.1)
\end{aligned}
$$

Similarly, since $\mathcal{L}(T_2) \neq \mathcal{L}(T_3)$,

$$
\begin{aligned}
& B(T_3, T_2) \\
= \; & |\mathcal{P}(T_2)| + |\mathcal{P}(T_3)| - \sum_{P \in \mathcal{P}} \min\left(|\mathcal{Q}'_{T_3}(P)|, |\mathcal{Q}''_{T_2}(P)|\right). \qquad (A.2)
\end{aligned}
$$

Combining (A.0) and (A.2), we obtain:

$$
\begin{aligned}
& B(T_1, T_3) + B(T_3, T_2) \\
= \; & |\mathcal{P}(T_1)| + 2|\mathcal{P}(T_3) \setminus \mathcal{P}(T_1)| + |\mathcal{P}(T_2)| - \sum_{P \in \mathcal{P}} \min\left(|\mathcal{Q}'_{T_3}(P)|, |\mathcal{Q}''_{T_2}(P)|\right). \\
= \; & |\mathcal{P}(T_1)| + 2|\mathcal{P}(T_3) \setminus \mathcal{P}(T_1)| + |\mathcal{P}(T_2)| \\
& - \sum_{P \in \mathcal{P}} \min\left(|\{Q' \in \mathcal{P}(T_3) : Q' \sim P\}|, |\{Q'' \in \mathcal{P}(T_2) : Q'' \sim P\}|\right). \quad (A.3)
\end{aligned}
$$

Since

$$
\begin{aligned}
& \sum_{P \in \mathcal{P}} \min\left(|\{Q' \in \mathcal{P}(T_3) : Q' \sim P\}|, |\{Q'' \in \mathcal{P}(T_2) : Q'' \sim P\}|\right) \\
\leq \; & \sum_{P \in \mathcal{P}} \min\left(|\{Q' \in \mathcal{P}(T_3) \setminus \mathcal{P}(T_1) : Q' \sim P\}|, |\{Q'' \in \mathcal{P}(T_2) : Q'' \sim P\}|\right) \\
& + \sum_{P \in \mathcal{P}} \min\left(|\{Q' \in \mathcal{P}(T_3) \cap \mathcal{P}(T_1) : Q' \sim P\}|, |\{Q'' \in \mathcal{P}(T_2) : Q'' \sim P\}|\right) \\
\leq \; & \sum_{P \in \mathcal{P}} |\{Q' \in \mathcal{P}(T_3) \setminus \mathcal{P}(T_1) : Q' \sim P\}| \\
& + \sum_{P \in \mathcal{P}} \min\left(|\{Q' \in \mathcal{P}(T_3) \cap \mathcal{P}(T_1) : Q' \sim P\}|, |\{Q'' \in \mathcal{P}(T_2) : Q'' \sim P\}|\right) \\
\leq \; & |\mathcal{P}(T_3) \setminus \mathcal{P}(T_1)| \\
& + \sum_{P \in \mathcal{P}} \min\left(|\{Q' \in \mathcal{P}(T_1) : Q' \sim P\}|, |\{Q'' \in \mathcal{P}(T_2) : Q'' \sim P\}|\right),
\end{aligned}
$$

by Eqn. (A.3) and (A.1),

$$
\begin{aligned}
& B(T_1, T_3) + B(T_3, T_2) \\
= \; & |\mathcal{P}(T_1)| + 2|\mathcal{P}(T_3) \setminus \mathcal{P}(T_1)| + |\mathcal{P}(T_2)| \\
& - \sum_{P \in \mathcal{P}} \min\left(|\{Q' \in \mathcal{P}(T_3) : Q' \sim P\}|, |\{Q'' \in \mathcal{P}(T_2) : Q'' \sim P\}|\right) \\
\geq \; & |\mathcal{P}(T_1)| + |\mathcal{P}(T_2)| \\
& - \sum_{P \in \mathcal{P}} \min\left(|\{Q' \in \mathcal{P}(T_1) : Q' \sim P\}|, |\{Q'' \in \mathcal{P}(T_2) : Q'' \sim P\}|\right) \\
= \; & B(T_1, T_2).
\end{aligned}
$$

The triangle inequality is proved. □

2 Proof of Proposition 7

Proposition 7 Let $k \geq 1$. The k-Bourque distance satisfies the following properties:

(1) For any 1-labeled trees S and T such that $|V(S)| = |V(T)| = n$, $B_k(S,T) = n \cdot B(S,T)$ if $k \geq \max(\text{diam}(S), \text{diam}(T))$, where $\text{diam}(X)$ is the diameter of X for $X = S, T$.

(2) $B_k(S,T)$ satisfies the non-negativity, symmetry and triangle inequality conditions.

Proof. (1). If $k \geq \max(\text{diam}(S), \text{diam}(T))$, $N_k(u) = S$ for any $u \in V(S)$ and $N_k(v) = T$ for any $v \in V(T)$. This implies that every edge has the same weight $B(S,T)$ and thus every perfect matching has a weight of $n \times B(S,T)$ in the graph $\text{BG}_k(S,T)$.

(2.) It is clear that $B_k(S,T)$ has the non-negativity and symmetry properties for each k. Let S, T and W be three 1-labeled trees. We assume that $s = |V(S)| \geq |V(T)| = t$ and consider three cases to prove that

$$B_k(S,T) \leq B_k(S,W) + B_k(W,T)$$

for $k \geq 1$.

Case 1. $w = |V(W)| \geq s$. Let us assume that:

$$f : \{v,\ \emptyset_i : v \in V(S), 1 \leq i \leq w - s\} \rightarrow V(W)$$

is a 1-to-1 function such that $\{(v, f(v)), (\emptyset_j, f(\emptyset_i)) : v \in V(S); 1 \leq i \leq w - s\}$ is a perfect matching with the minimum weight in $\text{BG}_k(S,W)$. Let us also assume that:

$$g : V(W) \rightarrow \{v,\ \emptyset_i : v \in V(T); 1 \leq i \leq w - t\}$$

is a 1-to-1 function such that $\{(v, g(v)) : v \in V(W)\}$ is a perfect matching with the minimum weight in $\text{BG}_k(W,T)$.

We now define the following:

$$\begin{aligned}
W_{00} &= \{v \in V(W) \ : \ v = f(\emptyset_i) \ \& \ g(v) = \emptyset_j\}, \\
W_{01} &= \{v \in V(W) \ : \ v = f(\emptyset_i) \ \& \ g(v) = t \in V(T)\}, \\
W_{10} &= \{v \in V(W) \ : \ v = f(s), s \in V(S) \ \& \ g(v) = \emptyset_j\}, \\
W_{11} &= \{v \in V(W) \ : \ N_k(v) = f(s), s \in V(S) \ \& \ g(v) = t \in V(T)\}.
\end{aligned}$$

Clearly, $|W_{10}| + W_{11}| = s$, $|W_{01}| + W_{11}| = t$ and $|W_{10}| - |W_{01}| = s - t$.

Let $W_{10} = \{a_1, a_2, \cdots, a_{k'}\}$ and $W_{01} = \{b_1, \cdots, b_k\}$, where $k' = k + s - t$. We then have:

$$\{(f^{-1}(v), g(v)) : v \in W_{11}\} \cup \{(f^{-1}(a_i), g(b_i)) : 1 \leq i \leq k\} \cup \{(f^{-1}(a_j), \emptyset) : k < j \leq k'\}$$

is a perfect matching in $\text{BG}_k(S,T)$ and its weight is:

$$\begin{aligned}
C \;=\; & \sum_{v \in W_{11}} B\left(N_k(f^{-1}(v)), N_k(g(v))\right) + \sum_{1 \leq i \leq k} B\left(N_k(f^{-1}(a_i)), N_k(g(b_i))\right) \\
& + \sum_{k+1 \leq i \leq k'} B\left(N_k(f^{-1}(a_j)), \emptyset\right).
\end{aligned}$$

Since the BD satisfies the triangle inequality,

$$\begin{aligned} & B\left(N_k(f^{-1}(a_i)), N_k(g(b_i))\right) \\ \leq\ & B\left(N_k(f^{-1}(a_i)), N_k(a_i)\right) + B(N_k(a_i), \emptyset) + B(\emptyset, N_k(b_i)) \\ & + B\left(N_k(b_i), N_k(g(b_i))\right) \end{aligned}$$

for any $1 \leq i \leq k$ and thus

$$\begin{aligned} C \ \leq\ & \sum_{v \in W_{11}} \left[B\left(N_k(f^{-1}(v)), N_k(v)\right) + B(N_k(v), N_k(g(v)))\right] \\ & + \sum_{1 \leq i \leq k} \left[B\left(N_k(f^{-1}(a_i)), N_k(a_i)\right) + B(N_k(a_i), \emptyset) + B(\emptyset, N_k(b_i))\right. \\ & \qquad \left. + B(N_k(b_i), N_k(g(b_i)))\right] \\ & + \sum_{k+1 \leq i \leq k'} \left[B\left(N_k(f^{-1}(a_j)), N_k(v)\right) + B(N_k(v), \emptyset)\right] \\ \leq\ & \sum_{v \in V(W)} B\left(N_k(f^{-1}(v)), N_k(v)\right) + \sum_{v \in V(W)} B(N_k(v), N_k(g(v))) \\ =\ & B_k(S, W) + B_k(W, T). \end{aligned}$$

By definition, $B_k(S, T) \leq C$, implying the triangle inequality.

Case 2. $t \geq w$.
Let us assume that

$$f : V(S) \to \{v,\ \emptyset_i : v \in V(W), 1 \leq i \leq s - w\}$$

is a 1-to-1 function such that $\{(v, f(v))\ :\ v \in V(S)\}$ is a perfect matching of the minimum weight in $\mathrm{BG}_k(S, W)$, and assume that

$$g : \{v,\ \emptyset_i : v \in V(W), 1 \leq i \leq t - w\} \to V(T)$$

is a 1-to-1 function such that $\{(v, g(v)), (\emptyset_i, g(\emptyset_i))\ :\ v \in V(W), 1 \leq i \leq t - w\}$ is a perfect matching of the minimum weight in $\mathrm{BG}_k(W, T)$. Then,

$$\begin{aligned} & \left\{\left(f^{-1}(v), g(v)\right)\ :\ v \in V(W)\right\} \cup \left\{\left(f^{-1}(\emptyset_i), g(\emptyset_i)\right)\ :\ 1 \leq i \leq t - w\right\} \\ & \cup \left\{\left(f^{-1}(\emptyset_j), \emptyset_{j-t+w}\right)\ :\ t - w < j \leq s - w\right\} \end{aligned}$$

defines a perfect matching in $BG_k(S, T)$ and its weight C can be bounded by:

$$\begin{aligned} C \ \leq\ & \sum_{v \in V(W)} \left[B\left(N_k(f^{-1}(v)), N_k(v)\right) + B\left(N_k(v), N_k(g(v))\right)\right] \\ & + \sum_{1 \leq i \leq t-w} \left[B\left(N_k(f^{-1}(\emptyset_i)), \emptyset_i\right) + B\left(\emptyset_i, N_k(g(\emptyset_i))\right)\right] \\ & + \sum_{t-w < j \leq t-w} B\left(N_k(f^{-1}(\emptyset_i)), \emptyset_{j-t+w}\right) \\ =\ & B_k(S, W) + B_k(W, T). \end{aligned}$$

Case 3. $s > w > t$. Let us assume that

$$f : V(S) \to \{v,\ \emptyset_i : v \in V(W), 1 \leq i \leq s - w\}$$

is a 1-to-1 function such that $\{(v, f(v)) \ : \ v \in V(S)\}$ is a perfect matching of the minimum weight in $\mathrm{BG}_k(S, W)$, and assume that

$$g : V(W) \to \{v, \ \emptyset_i \ : \ v \in V(T), 1 \leq i \leq w - t\}$$

is a 1-to-1 function such that $\{(v, g(v)) \ : \ v \in W\}$ is a perfect matching of the minimum weight in $\mathrm{BG}_k(W, T)$. Then,

$$\{(f^{-1}(v), g(v)), (f^{-1}(\emptyset_j), \emptyset_j) \ : \ v \in V(W), 1 < j \leq s - w\}$$

is a perfect matching in $\mathrm{BG}_k(S, T)$ and its weight is:

$$\begin{aligned} C &= \sum_{v \in V(W)} B\left(N_k(f^{-1}(v)), N_k(g(v))\right) + \sum_{1 \leq i \leq s-w} B\left(N_k(f^{-1}(\emptyset_j)), \emptyset_j\right) \\ &\leq \sum_{v \in V(W)} \left[B\left(N_k(f^{-1}(v)), N_k(v)\right) + B\left(N_k(v), N_k(g(v))\right)\right] \\ &\quad + \sum_{1 \leq i \leq s-w} B\left(N_k(f^{-1}(\emptyset_j)), \emptyset_j\right) \\ &\leq B_k(S, W) + B_k(W, T), \end{aligned}$$

where the inequality is derived from the triangle inequality. □

3 Measures for comparing mutation trees

3.0.1 The CASet and DISC metrics

The CASet and DISC metrics were introduced for mutation trees (DiNardo et al., Bioinformatics, 2020 Apr 1;36(7):2090-2097). Let M be a label set and T be a rooted tree in which the nodes are uniquely labeled with the parts of a partitions of M. For a node $u \in V(T)$, we use $\ell(u)$ to denote the label of u. For each $m \in M$, we use $\ell^{-}(m)$ to denote the unique node whose label contains m.

Recall that $A_T(u)$ denotes the set of ancestors of u and $u \notin A_T(u)$. For any $m \in M$, define $A_T(m) = \cup_{u \in A_T(\ell^{-}(m))} \ell(u)$. Note that $A_T(m') \cap A_T(m'')$ is equal to the set of their common ancestors for any m' and m'' of M.

Let S and T be two rooted labeled trees S and T whose nodes are uniquely labeled with the elements of M. The *Common Ancestor Set* (CASet) metric between S and T is defined as the average the Jaccard distance between the sets of common ancestors of two labels in S and T, i.e.,

$$\mathrm{CASet}(S, T) \triangleq \frac{1}{\binom{m}{2}} \sum_{i, j \in M : i < j} \frac{|(A_S(i) \cap A_S(j)) \triangle (A_T(i) \cap A_T(j))|}{|(A_S(i) \cap A_S(j)) \cup (A_T(i) \cap A_T(j))|},$$

where $A_S(i)$ is the empty set if i is not in the label set of S or it is an element of the label of the root of S. Here, the Jaccard distance between the empty set and itself is 0.

We use $D_S(i, j)$ to denote $A_S(i) \backslash A_S(j)$ for any two labels. The *Distinctly Inherited Set Comparison* (DISC) metric between S and T is defined to be [**?**]:

$$\mathrm{DISC}(S, T) \triangleq \frac{1}{m(m-1)} \sum_{i, j \in M : i \neq j} \frac{|D_S(i, j) \triangle D_T(i, j)|}{|D_S(i, j) \cup D_T(i, j)|}.$$

In a mutation tree, the nodes are labeled with disjoint subsets of the label set; a label appearing in a tree may not appear in another tree inferred for the same patient. It is not hard to generalize the CASet and DISC in the context of mutation trees.

3.0.2 An Ancestor Difference metric

One reason to introduce the Bourque distance is that every uniquely labeled tree can be uniquely reconstructed from all its node-induced star subtrees. It is not hard to see that every rooted uniquely labeled tree can also be reconstructed from the paths from the root to all other nodes. Hence, the difference between two mutation trees on M can be measured by the Ancestor Difference (AD) metric defined by:

$$\mathrm{AD}(S,T) \triangleq \frac{1}{|M|}\sum_{m\in M}\frac{|A_S(m)\triangle A_T(m)|}{|A_S(m)\cup A_T(m)|}.$$

The AD metric has been used for comparing mutation trees in (Govek et al, Proc. ACM BCB, 2018; Jahn et al., Genome Biology, 2016). Note that CASet, DISC and AD metrics do not satisfy the triangle inequality in general.

The triplet-based distance

The triplet distance has also been generalized to mutation trees (Coccolella et al., BioRxiv., 2020). In a mutation tree, any three labeled nodes induce a labeled tree that has three labeled nodes at most. The triplet-based distance (TD) between two mutation trees S and T with the same label set is defined by:

$$\mathrm{TD}(S,T) = 1 - \frac{|\mathrm{Triplets}(S)\cap \mathrm{Triplets}(T)|}{\max\left(|\mathrm{Triplets}(S)|, |\mathrm{Triplets}(T)|\right)},$$

where Triplets(S) denotes the set of possible subtrees induced by three different labels.

4 Pseudocode for computing Bourque distance between two labelled trees

Input : Labelled trees S and T, with $|E(S)| = s$ and $|E(T)| = t$ and label sets $\mathcal{L}(S)$, $\mathcal{L}(T)$
Output: $B(S,T)$
begin
  $C \longleftarrow \mathcal{L}(S) \cap \mathcal{L}(T)$ `// shared label set`
  if $C = \emptyset$ `// no shared labels`
  then
    return $s + t$ `//` $B(S,T) =$ `number of edges in` S `and` T
  end
  Remove labels in $(\mathcal{L}(S) \cup \mathcal{L}(T)) \setminus C$ from S and T
  Remove leafs in S with empty label set
  Root S at random node $r \in V(S)$ with $\ell(r) \neq \emptyset$
  Create mapping $m : C \to [1, ..|C|]$ based on node label accession order in pre-order traversal of S
  Relabel nodes of S based on m to obtain $\ell'(v)$ for all $v \in V(S)$
  $Z_S \longleftarrow \mathit{Partitions}(S, r, \ell')$ `// multi-set of intervals induced by subtrees`
  $\mathit{freq}_{Z_S}(I) \longleftarrow$ number of occurrences of I in Z_S for all $I \in Z_S$
  Relabel nodes of T based on m to obtain $\ell'(v)$ for all $v \in V(T)$
  Remove leafs in T with empty label set
  Root T at node r' with $1 \in \ell'(r')$
  $Z_T \longleftarrow \mathit{Partitions}(T, r', \ell')$ `// multi-set of intervals induced by subtrees`
  $\mathit{freq}_{Z_T}(I) \longleftarrow$ number of occurrences of I in Z_T for all $I \in Z_T$
  return $s + t - \sum_{I \in Z_S \cap Z_T} \min(\mathit{freq}_{Z_S}(I), \mathit{freq}_{Z_T}(I))$
end

Function `Partitions(`T, r, ℓ'`)`:
  $Z \longleftarrow \emptyset$ `// multi-set`
  for *each* $v \in V(T)$ *with* $v \neq r$ `// in post-order traversal`
  do
    $\mathit{lower}(v) \longleftarrow \min\{\min_{u \in C_T(v)} \mathit{lower}(u), \min_x \{x \in \ell'(u)\}\})$
    $\mathit{upper}(v) \longleftarrow \max\{\max_{u \in C_T(v)} \mathit{upper}(u), \max_x \{x \in \ell'(u)\}\})$
    $\mathit{count}(v) \longleftarrow \sum_{u \in C_T(v)} \mathit{count}(u) + |\ell'(u)|$
    if $\mathit{upper}(v) - \mathit{lower}(v) = \mathit{count}(v) - 1$ `// true if consecutive interval`
    then
      $Z \longleftarrow Z \cup [\mathit{lower}(v), \mathit{upper}(v)]$
    end
  end
  return Z

References

1. Govek K, Sikes C, Oesper L. A consensus approach to infer tumor evolutionary histories. InProceedings of the 2018 ACM International Conference on Bioinformatics, Computational Biology, and Health Informatics 2018 Aug 15 (pp. 63-72).

2. Jahn K, Kuipers J, Beerenwinkel N. Tree inference for single-cell data. Genome Biology. 2016 Dec;17(1):1-7.

3. Ciccolella S, Bernardini G, Denti L, Bonizzoni P, Previtali M, Della Vedova G. Triplet-based similarity score for fully multi-labeled trees with poly-occurring labels. BioRxiv. 2020 Jan 1.

4. DiNardo Z, Tomlinson K, Ritz A, Oesper L. Distance measures for tumor evolutionary trees. Bioinformatics. 2020 Apr 1;36(7):2090-7.
