# Supplementary material for: The Bourque distances for mutation trees of cancers
Source: Algorithms Mol Biol. 2021 Jun 10;16:9. doi: 10.1186/s13015-021-00188-3 (PMC8193869; doi:10.1186/s13015-021-00188-3)
Supplement: Supplementary file 2 — Additional file 2: Figure S1. Let T0 be the rooted star tree whose root is 1 and whose leaves are 2 to 7. A. Two rooted trees such that the ancestor difference measure between T0 and them are 0.1428, but the Bourque distance between T0 and them are 2 and 4. B. Two rooted trees such that the triplet-based distances between T0 and them are 0.3715, the common ancestor set measure between T0 and them are 0.0238, but the Bourque distance between T0 and them are 2 and 4. [file 13015_2021_188_MOESM2_ESM.pdf]

A B

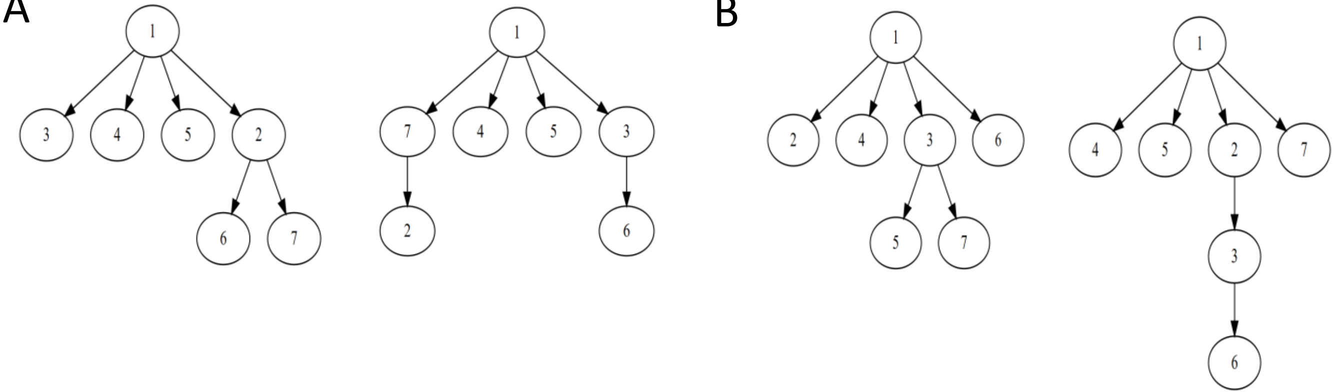


**Figure S1**. Let $T_0$ be the rooted star tree whose root is 1 and whose leaves are 2 to 7.
A. . Two rooted trees such that the ancestor difference measure between $T_0$ and them are 0.1428, but the Bourque distance between $T_0$ and them are 2 and 4.
B. Two rooted trees such that the triplet-based distances between $T_0$ and them are 0.3715, the common ancestor set measure between $T_0$ and them are 0.0238, but the Bourque distance between $T_0$ and them are 2 and 4.
