# Supplementary material for: The Bourque distances for mutation trees of cancers
Source: Algorithms Mol Biol. 2021 Jun 10;16:9. doi: 10.1186/s13015-021-00188-3 (PMC8193869; doi:10.1186/s13015-021-00188-3)
Supplement: Supplementary file 3 — Additional file 3: Figure S2. The scatter plots of the Bourque distance vs the other distance measures between a rooted 1-labeled tree and 19,999 random trees with different label sets or multi-labelled nodesrooted at the same node in the second dataset generated with higher probability that are given inthe main text. BD: Bourque distance; AD: Ancestor distance; CASet: Common Ancestor Set distance;DISC: Distinctly Inherited Set; MLTED: Multi-label tree edit distance; TD: Triplet-based distance. [file 13015_2021_188_MOESM3_ESM.pdf]

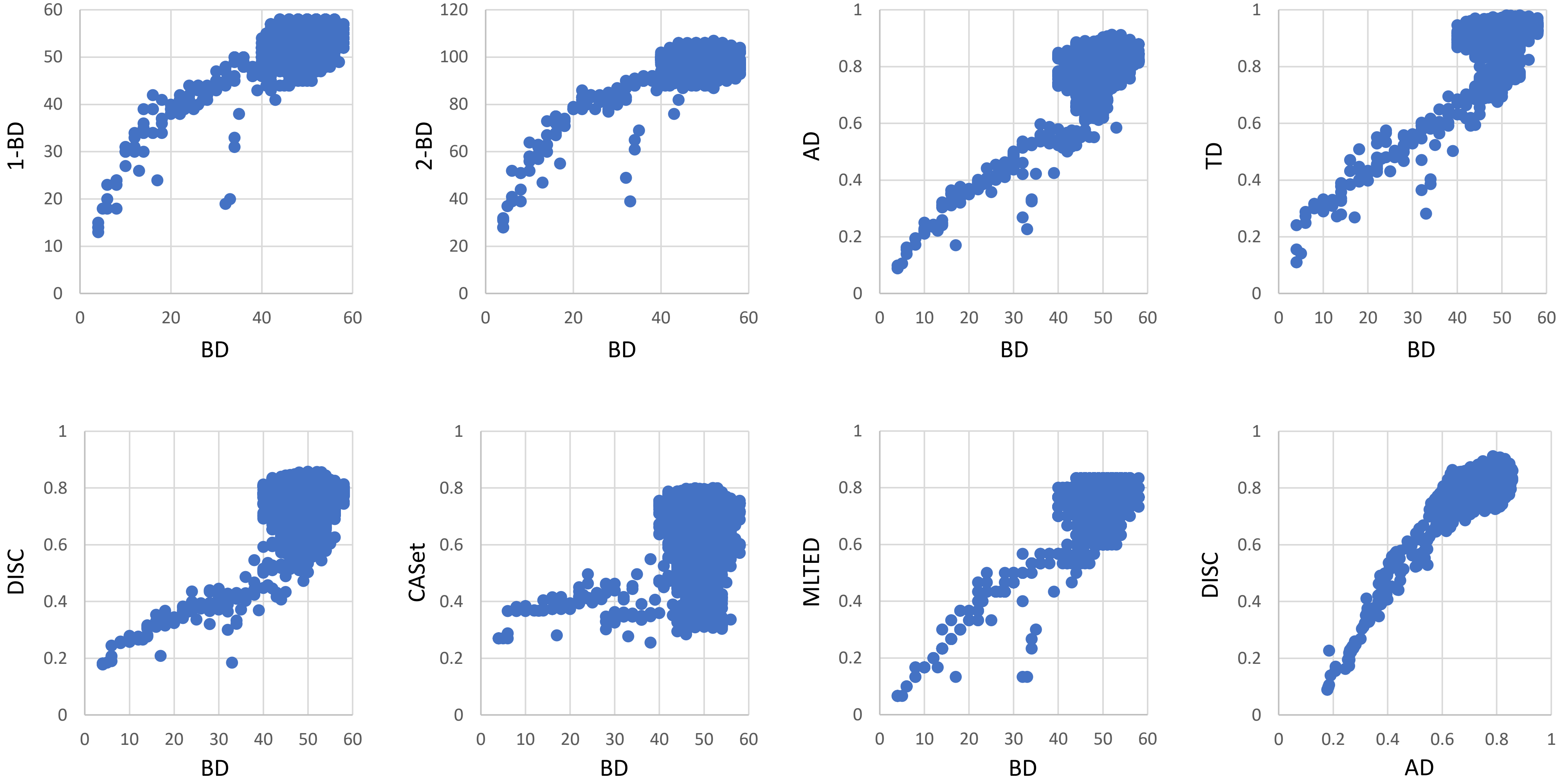

Figure S2. **The scatter plots of the Bourque distance vs the other distance measures between a rooted 1-labeled tree and 19,999 random trees with different label sets or multi-labelled nodes rooted at the same node in the second dataset generated with higher probability that are given in the main text.** BD: Bourque distance; AD: Ancestor distance; CASet: Common Ancestor Set distance; DISC: Distinctly Inherited Set; MLTED: Multi-label tree edit distance; TD: Triplet-based distance.
